# Towards Structural Systems Pharmacology to Study Complex Diseases and Personalized Medicine

**DOI:** 10.1371/journal.pcbi.1003554

**Published:** 2014-05-15

**Authors:** Lei Xie, Xiaoxia Ge, Hepan Tan, Li Xie, Yinliang Zhang, Thomas Hart, Xiaowei Yang, Philip E. Bourne

**Affiliations:** 1 Department of Computer Science, Hunter College, The City University of New York, New York, New York, United States of America; 2 Ph.D. Program in Computer Science, Biology, and Biochemistry, The Graduate Center, The City University of New York, New York, New York, United States of America; 3 Skaggs School of Pharmacy and Pharmaceutical Sciences, University of California San Diego, La Jolla, California, United States of America; 4 Department of Biological Sciences, Hunter College, The City University of New York, New York, New York, United States of America; 5 School of Public Health, Hunter College, The City University of New York, New York, New York, United States of America; National Cancer Institute, United States of America and Tel Aviv University, Israel, United States of America

## Abstract

Genome-Wide Association Studies (GWAS), whole genome sequencing, and high-throughput omics techniques have generated vast amounts of genotypic and molecular phenotypic data. However, these data have not yet been fully explored to improve the effectiveness and efficiency of drug discovery, which continues along a one-drug-one-target-one-disease paradigm. As a partial consequence, both the cost to launch a new drug and the attrition rate are increasing. Systems pharmacology and pharmacogenomics are emerging to exploit the available data and potentially reverse this trend, but, as we argue here, more is needed. To understand the impact of genetic, epigenetic, and environmental factors on drug action, we must study the structural energetics and dynamics of molecular interactions in the context of the whole human genome and interactome. Such an approach requires an integrative modeling framework for drug action that leverages advances in data-driven statistical modeling and mechanism-based multiscale modeling and transforms heterogeneous data from GWAS, high-throughput sequencing, structural genomics, functional genomics, and chemical genomics into unified knowledge. This is not a small task, but, as reviewed here, progress is being made towards the final goal of personalized medicines for the treatment of complex diseases.

## Introduction

Drug discovery, as broadly practiced, suffers from several shortcomings. First, although the vast amounts of genotypic and molecular phenotypic data generated from Genome-Wide Association Studies (GWAS); whole genome sequencing (WGS) [1]; and high-throughput techniques such as RNA-seq [2], ChIP-seq [3], BS-seq [4], and DNase-seq [5] provide an unprecedented opportunity to understand the etiology of complex diseases and to discover safe and potent personalized medicines, to date these data have not been fully explored to improve the effectiveness and efficiency of drug discovery. Second, modern target-based drug discovery is characterized as a one-drug-one-gene paradigm, and has been of limited success in attacking complex diseases. Third, phenotypic screens and cell-based assays generate a large number of active compounds relevant to disease treatment, but give few hints as to what their molecular targets are [6]–[9]. As a result of these shortcomings, the cost to launch a new drug is typically more than US$1 billion, and that cost continues to increase, with only around one-third of drugs in phase III clinical trials reaching the market. The emerging field of systems pharmacology is addressing these shortcomings and beginning to change the way we think about drug action in multigenic, complex diseases [10]–[15].

**Figure 1:**
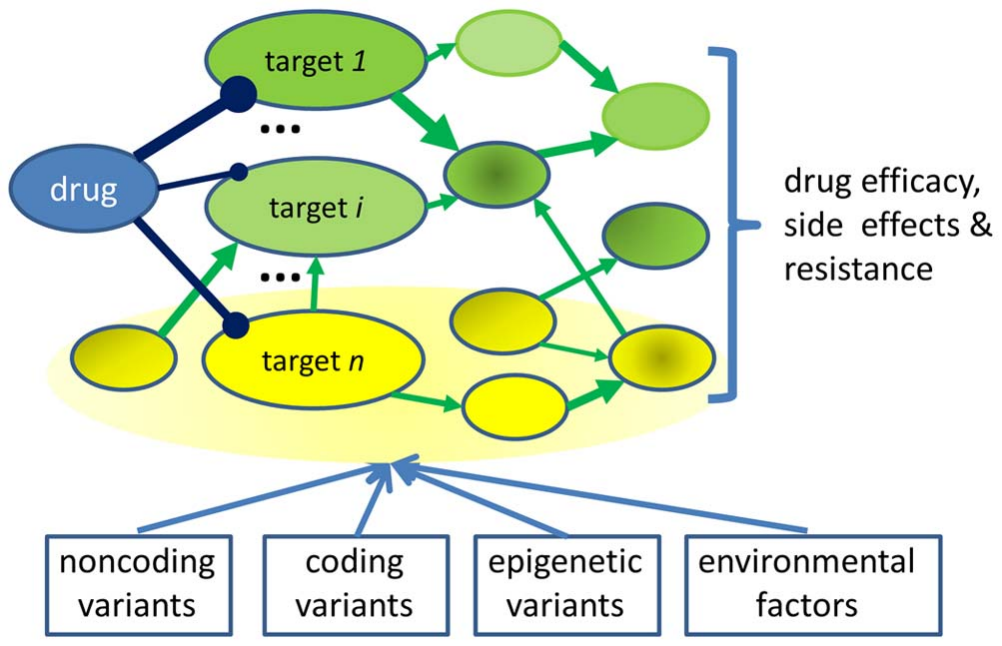
A network view of drug action. Dark blue lines represent drug–target interactions. Green arrows are protein–protein interactions or biological reaction pathways. Yellow nodes represent genes affected by genetic variation. These variations will impact drug action by changing the information flow of drug–target interactions in the biological network, even when these genes are not themselves the direct drug targets.

As illustrated in Figure 1, a drug commonly not only interacts with its intended molecular target (on-target) but also binds to and affects other targets (off-targets) that are often unknown [16]. Each drug–target interaction modifies the conformational dynamics of the target structure and results in the alternation of the functional states (e.g., activation versus inhibition). Consequently, the changing conformational and functional states of both on-targets and off-targets directly or indirectly affects other molecular components and their interactions through the interplay of complex signal transduction, gene regulation, and metabolic networks that collectively mediate the system-level response to the drug, leading to either therapeutic or adverse effects [10]. A variety of genetic, epigenetic, and environmental factors define the initial pathophysiological state of the molecular components and their interactions, which then dynamically evolve when perturbed by a drug. Stated another way, both target- and non-target associated genetic and/or epigenetic alternations could impact the drug response. In addition to inherited genetic and/or epigenetic factors, cellular, tissue, and organism environments may have significant effects on drug efficacy and side effects [17]–[21]. For example, tumor–stromal interactions play key roles in anticancer drug sensitivity [22].

The underlying hierarchical organization of living organisms makes it essential to model drug actions from DNA to gene, to protein and its molecular ensemble, to cell, to tissue, to organ, to whole organism, and to population. Data-driven, network-based association studies and physical- or mathematical-based multiscale modeling are two pillars of the existing paradigm of systems pharmacology. Network-based association studies provide a promising avenue to realize personalized medicine. The reconstruction and analysis of genome-scale molecular interaction networks, including protein–protein interactions; protein–nucleic acid interactions; epistasis interactions, as found in signal transduction; gene regulation; and metabolic networks, have emerged as a powerful framework to integrate heterogeneous DNA variation and omics data in associating genotypes with phenotypes under various environmental and drug-induced conditions [16]. By taking advantage of the progress in network and systems biology, mechanism-based multiscale modeling that spans different temporal and spatial scales has already been able to predict genotype-phenotype associations in a whole cell model of *Mycoplasma genitalium* [23] and quantitatively simulate drug actions at the organism level [24].

However, several challenges remain in the application of systems pharmacology. First, data-driven network-based association studies primarily rely on sophisticated statistical techniques. Although great efforts have been made to address the *n*≪*p* problem, where the number of observations *n* is much smaller than the number of variables or parameters *p*, the power of these statistics-based techniques remains limited if sample sizes are small. The “causal” relationships inferred from these methods are simply mathematical correlations. They may not provide biological insight into the underlying molecular mechanisms that enable the development of actionable models for understanding the drug-response phenotype. Second, the existing paradigm of multiscale modeling is to isolate subsystems (or parameters) from a phenotype space. These subsystems are first studied independently and then combined to infer their synergistic behavior. It is noted that the subsystem itself can be considered as a phenotype. Thus, this isolation/combination process could be multilevel and recursive. However, current organism-level physiological models are often too complex to be supported by existing data and computational power. The mathematical model is often nonidentifiable; that is, there is not a unique parameter set to explain the experimental observations [25]. On the other hand, physical models are often too computationally intensive to readily model the global behavior of the physiological system. Fundamentally, isolated parameter space may be not sufficient to “identify and elucidate the guiding principles of control and communication defining the behavior of an organism” [11]. Such a guiding principle is fundamental to reliably predict human behavior by scaling up animal models. Third, the enormous investment in molecular libraries and target-, cell-, and organism-based high-throughput compound screening has generated a massive amount of chemical genomics data [26]–[28]. There is no doubt that these data are invaluable in understanding how drugs work at the molecular, cellular, and organismal levels. However, this arguably most important dataset for systems pharmacology has not been fully incorporated into either the network-based association studies or multiscale modeling frameworks, partly due to a lack of computational tools to map bioactive chemical space to its global target and pharmacological space. Lastly, it has been recognized that one of the critical hurdles in multiomics data integration and multiscale modeling is the lack of a common language and standard to annotate, exchange, reuse, and update computational models [29]–[31]. Due to the dynamic, complex, and multiscale nature of datasets and computational models needed to simulate a drug response under diverse genetic, epigenetic, and environmental conditions, an open and reusable conceptual framework that is able to link multilevel biological concepts and relationships is needed to realize the promise of community-driven predictive modeling of human physiology and pathology [32]–[35].

Although macromolecular structure is at the foundation of any molecular interaction, adding the structural and associated energetics and dynamics of the interplay between drugs, biomolecular targets, genetic and epigenetic variations, and environmental factors has not been fully exploited in systems pharmacology to date. A global three-dimensional macromolecular structure view of the biological system under study may offer new insights to address the aforementioned challenges. Stated more explicitly, a mechanistic understanding of how individual molecular components work together in a system and how the molecular interactions are affected and adapted to genetic and epigenetic variants and environmental perturbations requires knowledge of the underlying molecular structures and their conformational dynamics [36]. The information derived from the atomic details of molecular interactions, in principle, will enhance the power of statistical inference in data-driven systems biology and alleviate the current inability to fully characterize parameter space in mathematical modeling, revealing the guiding principles of systematic control and communication. Moreover, as a bridge to connect chemical and genomics space, macromolecular structure will allow us to link drugs, targets, and biological pathways, thereby providing a common framework to correlate molecular interactions with cellular functions. *Leveraging the vast investment in chemical genomics, functional genomics, structural genomics, and structure-based drug discovery, together with efforts in systems pharmacology, may open a new door to developing personalized medicines for complex diseases*. Thus, here we advocate and then justify a new paradigm of structural systems pharmacology. Structural systems pharmacology will model, on a genome scale, the energetic and dynamic modifications of macromolecules (proteins, RNA, DNA) by drugs. The modeling accounts for genetic/epigenetic and environmental factors as well as the subsequent collective effects on the information flow in biological systems.

Some advances have been made in incorporating macromolecular structure modeling into systems pharmacology. We review them in this article. We demonstrate that integrative modeling of drug action—from the structural and energetic basis of genome-wide molecular interactions to the clinical outcomes at the organism level—provides new insights into both therapeutic effects and side effects while taking into account genetic differences. In terms of scope, we first propose a hybrid modeling approach to integrate mechanism-based multiscale modeling and simulation with a data-driven systems biology approach and suggest that macromolecular structure is an essential component to glue diverse technologies together into a unified framework. We demonstrate the potential of macromolecular structures in enhancing the capability of systems biology through enriching the connectivity of context-specific biological networks and resolving non-identifiable parameter space during network simulation. Then we focus on three aspects of structural systems pharmacology that link genetic events and drug–target interactions to the drug response phenotype. First, we focus on traditional pharmacodynamics, now in the context of structural systems pharmacology. Second, and in a similar fashion, we focus on traditional pharmacokinetics in the context of structural systems pharmacology. Third, we explore the role of structural systems pharmacology to enhance the power of pharmacogenomics and GWAS. Thus, this review complements several recent reviews that focus on a network view of systems pharmacology and its connection to phenotype [10]–[15]. Physical-based multiscale modeling will not be covered in detail, since this is presented elsewhere [37]–[40]. Ultimately, we argue, structural systems pharmacology should be incorporated into the modeling and simulation of macromolecular ensembles, tissues, and organisms.

## Structural Systems Pharmacology—Structure-Enabled Integrative Modeling of Drug Action

**Figure 2:**
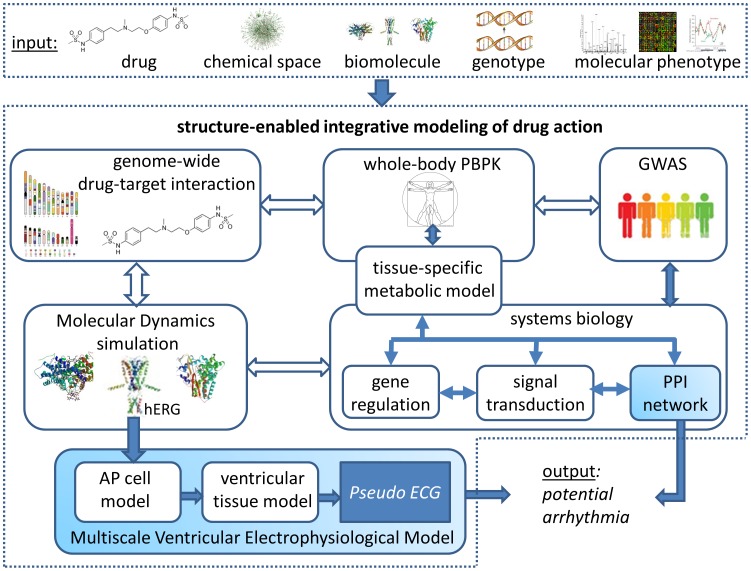
A structure-enabled integrative framework to model drug action. Given a set of inputs—a new or existing drug, known bioactive chemical space, the whole human proteome, an individual's genotypic data, and context-specific phenotypic data—it is possible, in principle, to construct a structure-enabled integrative model of drug action. Such a model comprises multiple integrated functional modules (rounded boxes) that span multiple levels of biological organization and can be used to infer drug-induced arrhythmia. Solid and open arrows indicate current workflows and missing links, respectively. Blue boxes represent two existing methods: multiscale ventricular electrophysiological modeling [41]–[42] and protein–protein interaction (PPI) network-based predictive modeling [43] for the prediction of drug-induced arrhythmia represented as a pseudo electrocardiography (ECG). The other boxes represent functional modules that are critically important but have not been fully developed or incorporated into the modeling process.

**Figure 3:**
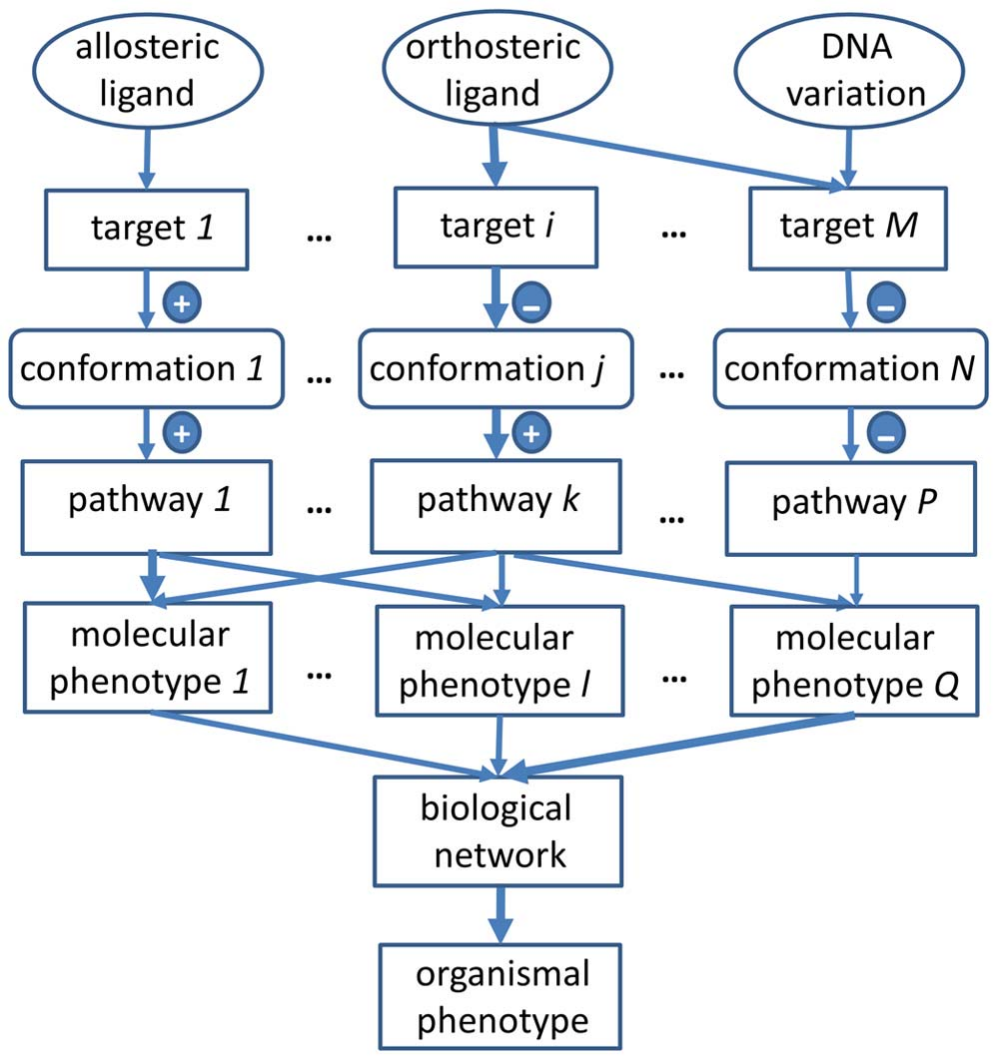
Hierarchical cause-effect semantic modeling to understand and predict drug action across temporal and spatial scales by using diverse techniques and integrating multiple sequencing, molecular, and omics data. Arrowed edges represent cause-effect relationships between biological entities: genetic variation, ligand (allosteric or orthosteric), drug target, conformational state of the drug target, biological pathway, molecular phenotypes from multiple omics data, integrated biological network, and organism phenotype (e.g., disease). The thickness of the arrow indicates the degree of probability. And the + and − signs represent positive (or activated) and negative (or inhibited) regulation, respectively. For example, an allosteric ligand may interact with target *1* to induce its active conformation that positively regulates pathway *1*. The positively regulated pathway *1* can be derived from an observed molecular phenotype *1* (e.g., gene expression profile). A context-specific biological network can be inferred by integrating multiple molecular phenotypes and be used to understand and predict an organismal phenotype.

As stated in the introduction, several challenges remain in systems pharmacology, limiting the ability to predictively model complex drug action under the influence of diverse genetic, epigenetic, and environmental factors. To address these challenges, we suggest an integrative structural systems pharmacology approach to understanding and predicting individual- and context-specific drug response phenotypes. In this proposed modeling framework, macromolecular structure is an indispensable component to link chemical space to genomic space, to associate genotypes with phenotypes, and to account for the environmental impact on biological systems. To demonstrate the proposed model, we use the predictive modeling of drug-induced arrhythmia as an example (Figure 2). Drug-induced arrhythmia is a potentially life-threatening side effect that is a major concern in clinical trials. QT interval prolongation that can be measured by electrocardiogram (ECG) waves has been widely accepted as a biomarker for arrhythmia. Both multiscale physical and mathematical models [41]–[42] and network-based predictive models [43] have been developed to predict QT interval prolongation with the aim of predicting the drug side effect of arrhythmia at an early stage (blue-colored box in Figure 2). However, several key components are missing in these models. As a result, their prediction power is limited. Given a new or existing drug, we at least need to address the following issues in order to predict whether or not the drug may induce arrhythmia under a specific physiological context for a specific individual (Figure 2):

1. Identification of genome-wide drug–target interactions. The QT interval prolongation involves not only multiple ion channels (e.g., hERG, Kv7.1, Nav1.5, and Cav1.2) but also multiple other genes that are functionally associated with the ion channel [43]. In addition, the interaction of a drug with metabolizing enzymes and regulatory genes may alter the concentrations of proteins that play roles in arrhythmia and the pharmacokinetic profile of the drug molecule.
2. Conformational dynamics and energetics of multiple ion channels under drug and genetic perturbation. The dynamic change of ion channel conformations (open and closed) during gating is the primary determinant of the membrane current during the action potential. Both drug binding and unbinding kinetics, as well as amino acid mutations, may impact the conformational change of the ion channel, leading to the change of action potential. The events of conformational dynamics can be modeled by Molecular Dynamics (MD) simulation. As genome-scale MD simulation is not feasible at this time, evolutionary and functional constraints that could be derived from sequencing and multiple omics data will be an invaluable asset to significantly reduce the conformational sampling space of MD simulation [44].
3. Determination of the in vivo concentrations of relevant drugs and metabolites (e.g., ebastine [45]) that may affect the activity of ion channels. In principle, this could be achieved using a whole-body physiologically based pharmacokinetics (PBPK) model that incorporates tissue-specific, genome-scale metabolic models. However, to date, little attention has been paid to adjusting PBPK models using information from genome-wide drug–target interactions.
4. Identification of individual- and context-specific parameter spaces for PBPK and systems biology models. To predict individual- and context-specific (e.g., normal tissue versus inflamed tissue) drug responses, it is critical to define molecular states, network architectures, and dynamic parameters at the molecular level under the physiological conditions that exist during drug treatment. Although a vast amount of GWAS and multiple types of omics data provide abundant opportunities for this purpose, these data have not been fully explored to define biological networks at molecular resolution.
5. Most importantly, it is necessary to integrate the above information into a coherent computational model across temporal and spatial scales. As these tasks traditionally span multiple disciplines and require different techniques, such as statistical machine learning [46], MD simulation, Ordinary Differential Equation (ODE)-based kinetics simulation, constraint-based modeling, discrete logic models, etc., they may need to be implemented as independent functional modules and subsequently assembled into a complete framework. Consequently, these functional modules need clearly defined interfaces and metadata for bi-directional communication. For example, the PBPK model determines the fate of drug molecules. In turn, drug–target interactions may regulate the expression level of CYP450 (as detailed later), thus altering the parameter space of the PBPK model. It has been suggested that ontology-driven, rule-based modeling may facilitate the integrative modeling of drug actions [11]. The integration of rule-based semantic modeling and Bayesian statistical modeling [47]–[49], which can establish cause–effect relationships across temporal and spatial scales, could be a useful tool in combining diverse techniques and multiple sequencing, molecular, and omics data. Such a scheme is depicted in Figure 3. It combines information and biological knowledge from DNA variants and their associated genes; drug–target interactions; protein conformational states; biological pathways; cellular networks, such as protein–protein interaction networks; molecular phenotypes, such as gene expression profiles; and different organism phenotypes. Using these individualized drug response phenotypes, the probability of causal mutations, involved targets, conformational states, molecular complexes or functional modules, and biological pathways and their links can be established by a priori knowledge from mechanism-based modeling or estimated using a Bayesian statistical framework.

Although we use the arrhythmia example for illustrative purposes, the framework described in Figure 2 should be generalizable for the understanding and prediction of drug response phenotypes. As detailed in the remaining text, we will show that macromolecular structures play critical roles in reconstructing genome-scale, high-resolution molecular interaction models, simulating the conformational dynamics of drug–target complexes, enabling context-specific pharmacokinetics modeling, resolving nonidentifiable parameters in mathematical modeling, and enhancing the predictive power of network-based association studies. Thus, the structure-enabled integrative modeling of drug actions may facilitate transforming conventional drug discovery process to a new paradigm based on systems pharmacology.

## Molecular Resolution of the Biological Network and Its Parameter Space

Reconstruction of individual- and context-specific genome-scale biological networks (e.g., drug–target, protein–protein interaction (PPI), metabolism, gene regulation, and signal transduction) is the foundation of systems pharmacology. Concurrently, protein structure-based PPI networks [50]–[55] have already made significant contributions to reliably expanding genome-scale PPIs [51]–[52], understanding the molecular mechanism of signal transduction [56]–[57], revealing the evolutionary origin of pathogen–host interactions [58], elucidating the molecular basis of disease mutations [59], and designing novel molecular therapeutics to target the network, PPI interfaces, and allosteric modulation, as summarized by Duren-Frigola et al. [60].

When kinetic parameters are lacking, constraint-based Flux Balance Analysis (FBA) presents an alternative approach to compute the phenotypic properties of whole cells, especially genome-scale metabolic networks [61]. New biological insights have been gained when incorporating protein structural information into metabolic network modeling of bacteria, which cannot be achieved by FBA alone [62]–[63]. For example, structure-based reconstruction of a genome-wide metabolic network makes it possible to determine bacteria growth in response to temperature changes [64]. This opens a new door to understanding the impact of the environment on drug action. It is noted that when protein structures are used, they are often treated as a single chain or as simply forming binary interactions during network analysis. In reality, under physiological conditions, proteins perform their functions through biological assemblies that may consist of multiple proteins. In a recent study, the 3-D structure of biological assemblies has been explicitly considered in the context of the genome-scale metabolic network. Novel drug targets and therapeutics are expected to be identified through such an integrative modeling strategy [65]. Beyond microorganisms, the first reconstruction of a genome-scale human metabolic network (Recon-1) by Duarte et al. provided the foundation for applying FBA to complex disease modeling [66]. By taking advantage of the rapidly increasing omics data, new methods have been developed to model cell-specific [67], tissue-specific [68]–[69], and context-specific [70]–[71] human metabolic networks.

**Figure 4:**
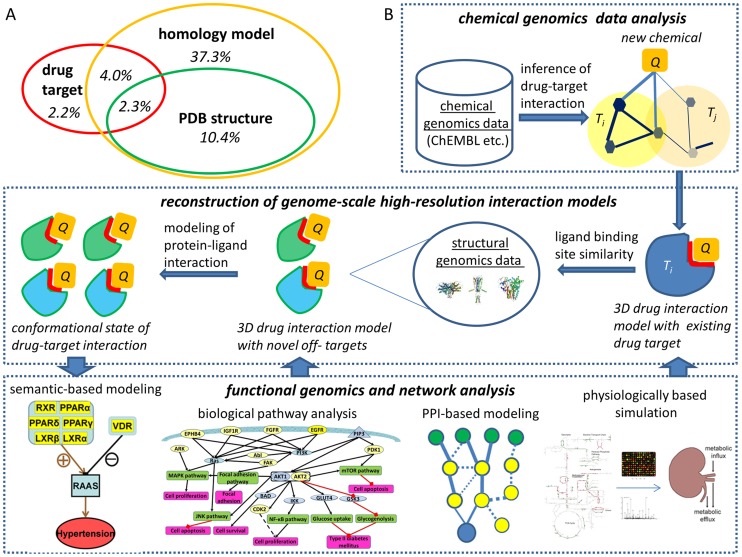
Reconstruction of genome-wide, high-resolution protein–chemical interaction networks. (A) Distribution of existing drug targets, PDB structures, and homology models in the human genome. (B) A schema to reconstruct 3-D drug–target interaction networks by integrating chemical genomics, structural genomics, and functional genomics. Novel drug off-targets could be identified by using the drug–target interaction models from chemical genomics analysis, and followed by searching for entire human or pathogen structural genome. In addition to sequence and global structural comparison, ligand binding site comparison is a valuable method, as it can identify binding promiscuity across fold space [119]–[121], [231]–[238]. After putative off-targets have been identified from structural genomics analysis, sophisticated molecular modeling techniques such as protein-ligand docking and Molecular Dynamics (MD) simulation can be applied to determine high-resolution interaction models and their binding affinity and conformational space. To correlate drug–target interactions with their physiological response, the conformational state of the drug–-target complex can be mapped to biological pathways, integrated networks, and physiological models. Several examples are shown in the figure. Semantic-based modeling is able to establish cause-effect from drug to target to pathways and, ultimately, to clinical outcomes [98]. Biological pathway analysis will provide the mechanistic understanding of information flow caused by drug modulation [44]. Critical components and interactions involved in drug modulation can be identified through integrated protein–protein interaction (PPI) network analysis [212]. Here, blue and green nodes represent drug targets and genes with observable changes, respectively. The target inhibition or activation along with genetic perturbations can be simulated using reconstructed physiological models [71]. In turn, the information from pathway and network analysis can be used to verify or falsify the drug–target interaction models and to constrain their conformational space.

Several recent efforts have made progress in reconstructing genome-scale high-resolution protein–chemical interaction models [72]–[76]. As shown in Figure 4A, the targets identified from chemical genomics and functional genomics data analysis mainly include existing known drug targets and their homologs, which only cover a small portion (∼8%) of the human genome [77]–[95]. A large number of proteins whose cognate or designed ligands are less characterized (or unknown) or who have low-affinity bindings to drugs are very likely to be important or even critical to pathophysiological processes under consideration [16]. The target space can be significantly extended to ∼50% of human genes using structural genomics data [96]. As illustrated in Figure 4B, when integrating chemical genomics data analysis and molecular modeling on a structural genome scale, it is possible not only to greatly extend the existing target space to ∼50% of human and pathogen genomes (a five to 50 times increase over existing targets), but also to construct genome-wide high-resolution protein–chemical interaction models for millions of bioactive compounds [72], [97]. Although it remains a challenge to accurately determine, under physiological conditions, the binding affinity and binding/unbinding kinetics of these interaction models, these models provide a basis to simulate the conformational dynamics of protein targets perturbed by a drug (see details in next sections) [44], [98]. Consequently, the physiological drug response (therapeutic effect or side effect) can be predicted by mapping the conformational states of drug targets into biological pathways and networks [44], [98]. We expect that the integration of chemical genomics, structural genomics, and functional genomics will significantly enhance the capability of systems pharmacology for molecular target identification of bioactive compounds, drug repurposing, polypharmacological drug design, and side effect prediction.

In the context of personalized medicine in the treatment of complex diseases, a critically important but less-addressed problem is the need to reconstruct genome-scale, structure-based gene regulatory networks. In the major groove of DNA, every base pair has a unique hydrogen-bonding signature, and the “direct readout” mechanism, in which the formation of a series of amino acid base-specific hydrogen bonds contributes to the protein-DNA binding specificity, has been commonly accepted [99]. Recently, Rohs et al. found that the binding of arginine residues to narrow minor grooves is a widely used mode for protein–DNA recognition [100]–[101]. Differing from the “direct readout” mechanism, their findings indicate that the minor groove can also provide information, such as local variations in DNA shape and electrostatic potential for protein–DNA recognition, and offer new insights into the structural and energetic origins of protein–DNA binding specificity. These studies highlight the importance of the role of macromolecular structures in understanding gene regulation.

The reconstruction of the biological network is only the first step in understanding the dynamic and stochastic nature of cellular processes [102]. +When the network topology and kinetic parameters are defined, time-dependent deterministic functions, including Ordinary Differential Equations (ODEs) and Partial Differential Equations (PDEs) [103], are commonly employed to analyze the dynamics of signaling and metabolic pathways upon drug treatment [104]. For instance, ODEs have been successfully applied to investigate the effects of multitarget inhibition [105]–[106], to search for optimal target combinations for safe and effective anti-inflammation therapy [107], and to predict the network response to target inhibition [108]. PDEs can be used to model the concentration change of each component as a function of both time and space [103]. Such a technique is important in determining the effective concentration of a drug when it reaches its targets and off-targets in an essentially nonlinear, non-equilibrium cellular and microenvironment. Stochasticity is one of the fundamental properties of cellular processes [102]. Moreover, in vivo drug action involves a series of stochastic processes such as prodrug transport and efflux [109]. Thus, stochastic models will be important tools in modeling drug action [110].

However, dynamic modeling is hampered by the lack of reliable kinetic parameters. In many cases, the kinetic parameters for enzyme reactions can be estimated from protein structures [111]. The computational techniques required are dependent on the reaction mechanism. Quantum Mechanics (QM) or Quantum Mechanics/Molecular Mechanics (QM/MM) is needed if bond-breaking is the rate-limiting step. Whereas, Brownian dynamics is more efficient if the reaction is fast and the diffusion rate is the determinant. Nevertheless, these computational techniques are time-consuming and cannot be easily extended to a genome scale. It has been proposed that the electrostatic potential in the active site determines not only the stability of transition states [112] but also the diffusion association rate [113]. Thus, it is proposed that the comparison of the Molecular Interaction Field (MIF), including the electrostatic potentials and other physical characteristics of structurally similar proteins, may assist in the estimation of the kinetic parameters [114]. A similar strategy has been applied to predict the association/disassociation rate of protein–protein interactions [115]–[117], which are essential for the dynamic modeling of signal transduction pathways. Furthermore, it is possible to extend the scope of MIF to a structural proteome scale for structurally unrelated proteins by analyzing and comparing the evolutionary, dynamical, physiochemical, geometric, and transition-state binding properties of the binding interfaces [118]–[122]. The use of estimated kinetics parameters is particularly promising when coupled with a kinetic hybrid model [123]. In these models, detailed rate equations are only used to describe essential enzymes. Simplified and approximate rate equations are applied to the majority of enzymes. A recent study has shown that 37% of enzymes in *Escherichia coli* are promiscuous and evolutionarily retained and catalyze 65% of known metabolic reactions [124]. With higher catalytic capability, specific enzymes tend to be essential and more frequently coupled with gene regulation than promiscuous enzymes. This finding provides additional support for the structure-based hybrid model. It is noted that the kinetic parameters are often experimentally measured under three-dimensional conditions, which do not reflect the two-dimensional dynamic processes occurring when a drug binds to a receptor. Wu et al. presented a theoretical multiscale simulation approach that converts three-dimensional affinities to two dimensions, accounting directly for the structure and dynamics of the membrane-bound molecules [125]. In summary, multiscale mathematical modeling and network-based association studies in systems pharmacology will benefit from the information derived from macromolecular structures in terms of the identification of reliable network connectivity as well as the enrichment of individual- and context-specific kinetic parameters.

### Pharmacodynamics in the Era of Structural Systems Pharmacology

A major focus of pharmacodynamics is to quantitatively understand drug–target interactions and their effects on the whole organism. It is clear that drug action cannot be fully understood by a conventional one-drug-one-target paradigm. A systematic view of all proteome drug–target interactions is often necessary [16]. A drug molecule may act more than to inhibit or activate a target in a binary manner. Recent studies in biased agonism (or biased signaling, functional selectivity) [126] and partial agonism add a new dimension to understanding pharmacodynamics. For example, it has been recognized that a G-protein coupled receptor (GPCRs) pleiotropically regulates multiple signaling pathways. An endogenous or designed agonist for a GPCR may selectively activate one of its regulated pathways, leading to therapeutic efficacy or, alternatively, to unwanted side effects. At the molecular level, biased agonism originates from the selection of specific conformational states of the target protein, which are dynamically coupled with ligand binding. Fundamentally, the molecular mechanism of biased agonism may be similar to that of partial agonism observed in nuclear receptors (NRs). The transcriptional activity modulated by NR agonists is not dependent on the binding affinity but rather the ensemble of both protein conformations and ligand orientations [127]–[128]. Moreover, allosteric interaction can shift the conformational ensembles, thereby modulating the activity of agonist binding [36].

These findings provide both new opportunities and impose challenges in linking in vitro drug binding with associated in vivo activity. In addition to identifying proteome-wide drug binding promiscuity and specificity, it is necessary to sample the conformational ensemble associated with these drug–target interactions and to link their conformational state with biological pathways. Although a number of state-of-the-art computational techniques (e.g., those reviewed in [16] and Figure 4) are able to predict drug binding cross-reactivity, few of them provide a high-resolution landscape for the complete conformational space of drug–target interactions. State-of-the-art methods accounting for conformational flexibility are not capable of mapping the conformational ensembles to the signaling pathways that they modulate [129]. New concepts and techniques are needed to include the influence of protein dynamics on functional activity of drug binding in the context of biological networks.

At the molecular level, conventional single-target, single-state virtual screening and quantitative structure-activity relationships (QSAR) should be extended to a multitarget, multiconformation model. A wide array of experimental techniques, such as fluorescence spectroscopy [130], plasmon waveguide resonance spectroscopy [131], bioluminescence resonance energy transfer [132], circular dichroism [133], X-ray crystallography [134], site-directed mutagenesis [135], and 19F-NMR spectroscopy [136], have been developed to determine active conformations associated with biased and partial agonism. These data provide a foundation for developing multistate models of pharmacodynamics. Structure-based molecular modeling may play a critical role in understanding the biased signaling. For example, dynamic protein-ligand homology modeling coupled with site-directed mutagenesis data is used to determine the dimerization and activation models for GPCRs [137]–[138].

In the context of personalized medicine, the functional selectivity of drug binding can be modulated by mutations in the protein target, which directly impact orthosteric or allosteric interactions. Thus, it is important to assess the importance of the mutation on the energetics and dynamics of proteome-scale drug–target interactions. Protein structure may provide critical insights into how the mutation alters the drug response. The genetic predisposition to adverse drug reactions (ADR) can be rationalized through the atomic details of the interaction between the drug and its potential off-target using structural modeling. For example, Li et al. have discovered that an R41Q mutation in human cytosolic sialidase (HsNEU2), which is predisposed in a small portion of the Asian population, links ADRs to oseltamivir (Tamiflu) [139]. In addition to mutations that directly involve drug binding, the mutations that disrupt allosteric interactions are some of the major determinants of disease but have been little studied [140]. Several structure-based techniques have been developed to predict the effect of mutations on allosteric regulation. They include correlated mutation analysis [141]–[146], the detection of pairwise dynamic [147]–[148] or energetic coupling [149] between residues, and analysis of the global topology of protein structures [150]–[151]. These methods may eventually contribute to the design of allosteric drugs [36], [152].

Dynamic simulation of drug unbinding kinetics is another area that may significantly impact personalized medicine. Drug–target interactions in vivo are different from those in vitro. In target or cell-based assays, the concentrations of both drug and target are fixed and the binding affinity is measured by thermodynamic equilibrium constants such as IC_50_ values, which reflect binding potency. However, in a living organism, the concentration of the drug, the target, and the other molecules constantly changes with time, rarely reaching equilibrium. Thus, the drug binding affinity is not an appropriate indicator of drug potency in vivo [153]. An increasing body of evidence suggests that drug efficacy correlates more strongly with drug–target residence time than with binding affinity [154]–[157]. Long residence time can lead to sustained pharmacological effect and may also alleviate off-target toxicity. The residence time of a drug on its target can be greatly influenced by conformational adaptation [158]. Recent studies suggest that the in vivo duration of drug efficacy not only depends on macroscopic pharmacokinetic properties like plasma half-life and the time needed to equilibrate between the plasma and the effect compartments, but is also influenced by long-lasting target binding and rebinding [159].

Experimental approaches to studying drug binding and unbinding to proteins have limitations in temporal and spatial resolution. It was reported recently that a computational network analysis combined with explicit water MD simulations of the unbinding of small inhibitors from the enzyme FK506 Binding Protein (FKBP) provided a clear picture of the free energy landscape (both thermodynamics and kinetics) of ligand dissociation [160]. The dissociation kinetics were characterized as a simple (i.e., single-exponential) time dependence with multiple dissociation pathways. A computational methodology using trajectory data from multiple Brownian dynamics simulations of ligand diffusion has been developed for characterizing the kinetics of drug–receptor interactions in terms of the encounter complex [161]. A computational approach named metadynamics has been used both for reconstructing the free energy and for accelerating rare events in systems described by complex Hamiltonians, at the classical or at the quantum level [162]. All-atom metadynamics simulations of a peptide substrate interacting with wild-type HIV-1 protease in explicit solvent rendered accurate calculations of binding affinity and kinetics constant compared to the experimental data [163].

Ultimately, drug–target interactions and genetic events should be studied in the context of biological networks. Existing biological network analysis is conformationally stateless. Thus, these networks are not sufficient to model the influence of protein dynamics on the drug response phenotype. The integrative modeling depicted in Figures 2 and 3 provides a possible solution to incorporating conformational dynamics into network modeling. It is worth mentioning that this integrative modeling framework could also be a powerful tool in studying the pharmacodynamics of drug–drug interactions. Due to the robust nature of biological systems, drug combinations are often necessary and proven to be successful in treating complex diseases and combating drug resistance [164]–[165]. However, the Adverse Drug Reaction (ADR) resulting from drug–drug interaction is a serious problem in developing combination therapies. In addition to the pharmacokinetics (see next section), the pharmacodynamics of the drug–drug interaction may play a critical role in the ADR [166]. Existing state-of-the-art methods for the prediction of drug–drug interactions are data-driven, which mainly establish statistical association from empirical observations but provide little information on the mechanism of drug–drug interaction. Thus, they may not be sufficient for the predictive modeling of drug–drug interactions during the early stages of drug development [167]–[168]. Using the proposed integrative modeling framework, it may be possible to predict drug–drug interactions de novo.

## Pharmacokinetics in the Era of Structural Systems Pharmacology

The Absorption, Distribution, Metabolism, and Excretion (ADME) properties of a drug, i.e., absorption and distribution to its target(s), detoxification by metabolism and excretion of the drug from the human body, are the primary concerns of pharmacokinetics. At the molecular level, ADME properties are strongly influenced by the abundance and activity of transporters and metabolizing enzymes such as CYP450, UDP-glucuronosyltransferases, sulfotransferases, N-acetyltransferases, glutathione S-transferases, and methyltransferases. Much effort has gone into developing computational methods for in silico prediction of ADME properties. These methods initially only addressed the small drug molecule. Based on chemical structures, quantitative structure-activity relationship (QSAR)-based approaches have been extensively used to correlate the physiochemical properties of lead molecules with their ADME profiles [169]–[170]. Shifting from the ligand to the receptor, structure-based methods have been developed which leverage the ever-increasing number of 3-D structures of ADME related proteins. Similarity searches and traditional pharmacophore approaches are enhanced by more advanced molecular descriptors and 3-D pharmacophores that encode the details of ligand-target binding [171]. Dynamic properties of ligand-target binding could be incorporated into the pharmacophore with conformational sampling techniques. Protein-ligand docking based on virtual screening for millions of compounds can now be accomplished with ease [172]. However, applying molecular docking to ADME related proteins is complicated by the existence of large and flexible binding cavities in CYP450 and phase II metabolizing enzymes, which can accommodate more than one ligand [172]. Consequently, the correlation between the docking score obtained for the best poses with experimentally determined binding free energies is usually poor. Nevertheless, in a recent study, Schlessinger et al. used a homology model of a norepinephrine transporter and molecular docking to successfully predict the prescription drugs which specifically bind to it [173]. With the availability of data from chemical genomics and high-throughput screening [28], [174], the combination of multiple flexible docking tools with chemoinformatics may boost the performance of structure-based virtual screening [175]. Although more accurate in deriving binding free energy, a more rigorous thermodynamic approach is unfortunately more computationally demanding and not applicable to large-scale virtual screening. Besides molecular docking based virtual screening or molecular dynamics simulation, quantum mechanical and hybrid quantum mechanical/molecular mechanical (QM/MM) methods have emerged as powerful tools for modeling reaction rates of drug metabolism. The whole reaction profile for benzene hydroxylation by CYP2C9 was studied with such a hybrid approach; a combined docking-MD-QM calculation was used to simulate the activation energy of CYP3A4 [176]. The challenge is to extend this technique to a structural proteome scale, as discussed in the previous sections.

**Figure 5:**
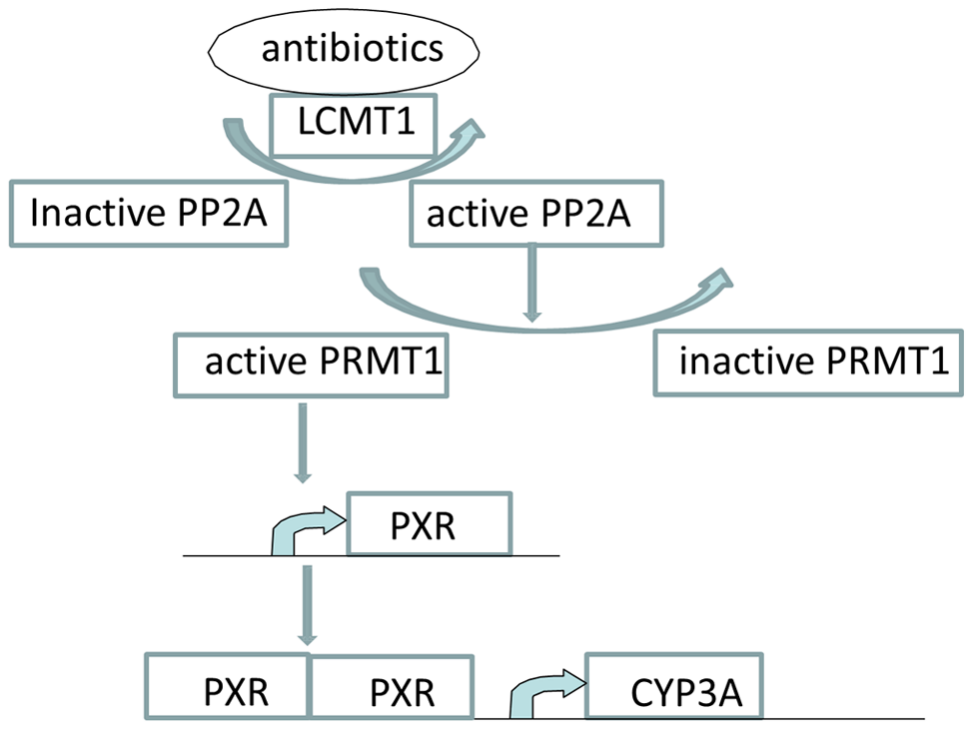
A proposed pathway that modulates the abundance and activity of CYP3A. LCMT1 is a potential off-target for antibiotics. The inhibition of LCMT1 will activate PXR, thereby increasing the activity of CYP3A.

So far, pharmacogenomics prediction of ADME properties has mainly focused on the genotypic variations and polymorphisms in metabolizing enzymes—the overall contribution of pharmacogenomics to personalized medicine remains limited [177]. The pharmacokinetics of a drug is the interplay between the inherent physiochemical properties of the drug and its physiological environment. The expression of metabolizing enzymes and transporters is highly regulated by multiple factors, including genetic polymorphisms, xenobiotics induction, cytokines, hormones, and pathophysiological states, as well as gender and age of families [178]. Multi-allelic genetic polymorphisms depend significantly on ethnicity and imply disparate clinical phenotypes including ADR, drug efficacy, drug resistance, and dose requirement. A mechanistic understanding of these regulators is essential for the predictive modeling of pharmacokinetics. It is well established that CYP genes are directly regulated by nuclear receptors [179]. Multiple genes, such as p53, AP-1, Ras, and APC, are involved in the regulation of multiple drug-resistance transporters (ABC transporters) [180]. If a drug itself, or another drug, interacts with these genes, undesirable pharmacokinetics profiles or drug–drug interactions may arise. Thus, the pharmacokinetics regulatory genes are potential drug off-targets that affect the fate of drug molecules in vivo. We call them “pharmacokinetics off-targets” to distinguish them from those related to pharmacodynamics. The fact that the activity of direct pharmacokinetics regulatory genes can be modified by their upstream genes adds a new layer of complexity to the problem. Therefore, to fully understand the molecular mechanisms underlying the ADME properties of a drug, it is necessary to identify the pharmacokinetics off-targets as well as the regulatory network of pharmacokinetics genes on a proteome scale. Figure 5 shows a regulatory pathway of CYP3A, whose substrates include several hundred drugs [181]. LCMT1 is a methyltransferase that methylates the PP2A catalytic subunit and promotes its functional association with the PP2A regulatory subunits. PP2A is a major protein phosphatase that dephosphorylates PRMT1, thus inhibiting PRMT1 enzymatic activity. PRMT1 is essential for the PXR transcription process. PXR dimerises with RXR to induce the gene expression of CYP3A. It is clear that genetic variations or drug perturbations on any one of the genes along this pathway may affect the abundance and activity of CYP3A, thereby leading to a change in the ADME properties. Using a structure-based off-target identification pipeline, LCMT1 has been identified as the off-target of several antibiotics [182], highlighting the potential power of proteome-scale structural modeling in predicting novel pharmacokinetics profiles and drug–drug interactions.

Noncoding DNA may play important roles in the regulation of transporters and metabolizing enzymes. For example, the CYP family includes 58 pseudogenes that do not encode functional protein [183]. An increasing body of evidence suggests that pseudogenes have diverse functions that influence not only their parent genes but also apparently unrelated genes [184]. For example, one of the CYP450 genes, CYP2A6, has a pseudogene, CYP2A7. CYP2A7 may transfer a fragment of DNA to its parent gene CYP2A6, leading to a change in its sequence. It is observed that individuals who smoke have a mutated gene CYP2A6*1B that is converted from a CYP2A7 polymorphism. CYP2A6*1B stabilizes its mRNA, thereby increasing first its expression level, then its activity in metabolizing nicotine [185]. The functional roles of most pseudogenes still remain elusive. Structural systems biology, as discussed in the next section, may shed new light on the functional relevance of noncoding DNA in pharmacokinetics.

Drug metabolism is strongly dependent on the physiological state (e.g., obesity and diabetes) and environment (e.g., gut-microbiome). The prediction of inter-individual pharmacokinetics variation requires the coupling of pharmacogenomics and pharmacometabonomics [186]. In principle, mechanism-based modeling of drug interactions with transporters and metabolizing enzymes can be integrated with pharmacogenomics, pharmacometabonomics, and other omics data using the integrative modeling framework proposed in the previous section. Physiologically based pharmacokinetics modeling has been developed in the past four decades. Although it is resource consuming in obtaining input parameters, progress made in in silico technologies has greatly facilitated the prediction of oral absorption and hepatic metabolism as well as mechanistic models of tissue distribution based on pharmacokinetics models. The bottleneck for physiologically based pharmacokinetics modeling resides in the limited data available. As discussed in the previous section, dynamic simulations involving comprehensive metabolic pathways, signaling pathways, and cell physiology are being studied together with multiscale modeling at the cellular, organ, and whole-body levels [187]. The sparse number of available kinetic parameters calls for more structure-based modeling efforts in order to enable further multiscale systems pharmacology analysis.

## Pharmacogenomics and GWAS in the Era of Structural Systems Pharmacology

Recent advances in pharmacogenetics and pharmacogenomics have identified genetic variants in several hundreds genes, notably drug metabolizing enzymes (e.g., CYP450), transporters, and drug targets [188]. The knowledge derived from such data has already resulted in individualized therapy. For example, the appropriate initial dose of the anticoagulation drug warfarin can be estimated using a pharmacogenomics algorithm [189]. Similarly, certain mutations can be used to predict alternative responsiveness to drugs in cancer therapy [190]–[194]. Acknowledgment of the emerging role of pharmacogenomics can be found in the labels of the Food and Drug Administration (FDA)-approved drugs montelukast [195] and cetuximab [196] and in FDA-approved diagnostic tests, for example, the microarray-based Roche AmpliChip for cytochrome P450 polymorphisms and the Invader UGT1A1 Molecular Assay for detecting polymorphisms that increase the risk of neutropenia when using the colon cancer drug irinotecan [15], [197].

In spite of these advances, much more is needed for predictive modeling of individual drug responses. It is critical to understand the impact of genetic variation beyond direct drug interactions with on-targets, off-targets, metabolizing enzymes, and transporters. Many nontarget-associated genetic factors may affect the drug response phenotype. As shown in Figure 1, if a critical node, edge, or feedback loop is modified in a drug modulation pathway, the drug efficacy or side effect profile can change accordingly. One example is the mutation of K-RAS located downstream of the EGFR pathway, which causes resistance to the anticancer activity of EGFR inhibitors [198]–[199]. In another situation, genes aside from the drug target may regulate the same biological pathway where the system-level drug response is the result of their combinatorial control. The mutation or expression changes of such genes may enhance or reduce the drug response. The gain- or loss-of-function of mutations that are associated with drug action may come from genetic variations in both coding and noncoding regions. Recently, the 1,000 Genomes Project has identified 38 million single nucleotide polymorphisms (SNPs), 1.4 million short insertions and deletions, and more than 14,000 larger deletions from 1,092 individuals belonging to 14 ethnic groups. The individual-specific, rare, coding variants are located across a broad array of biological pathways. Moreover, there are hundreds of functionally annotated, rare, noncoding variants for each individual [200]. It is expected that these variants will alter the pharmacokinetics, pharmacodynamics, and responsiveness to drug therapy [15]. The extremely large, multidimensional datasets from these studies present an exciting opportunity to expand the horizon of pharmacogenomics by identifying causal variants and genes, and predicting pathways likely to be involved in drug response. The challenge is how to associate these data with a predicted drug response a priori.

Many disease-associated variants and drug-response cryptic genetic factors remain uncharacterized; hence, new methods are urgently needed to annotate DNA variants, their functional roles, and their associations with drug actions. How to annotate the functional roles of DNA variants, especially for noncoding variants, is a challenge because of the diversity of noncoding functions, the incomplete annotation of regulatory elements, and unknown mechanisms of regulatory control. Several large-scale studies have been performed to annotate the noncoding genome and regulatory elements. These studies integrate the high-throughput functional genomics and comparative genomics datasets [3]–[5] to map the functional noncoding elements on a genome-wide scale. Variants on these elements can result in the different regulation of their target genes. For example, studies from the Encyclopedia of DNA elements (ENCODE) [201] and model organism Encyclopedia of DNA elements (modENCODE) [202]–[204] projects provide comprehensive maps of transcription binding for select cell lines and DNase maps for many primary cells and highlight the importance of noncoding DNAs in the regulation of complex phenotypes. With known functional elements and motifs, methods have been developed to predict the effect of newly observed rare and private mutations by integrating models of sequence motifs, chromatin states, and expression patterns in model organisms and in cultured human cells [205]–[208]. For example, quantitative sequence-activity models (QSAMs) [209] are trained based on these data in a massively parallel reporter assay (MPRA) developed to enable systematic dissection and optimization of transcriptional regulatory elements [208].

Since annotated pharmacogenomics biomarkers are incomplete and biased, the conventional “guilt-by-association” approach may not be sufficient to identify novel drug-response genetic markers [43]. The power of statistical analysis of pharmacogenomics and GWAS data is often limited by the moderate effect size of samples. As a result, a number of rare variants could be missed. Fundamentally, the genotype–phenotype relations established by statistical or machine learning approaches may be merely mathematical correlations. Macromolecular structures and their interactions may provide critical mechanistic insight into the functional roles of DNA variants and their impact on drug action. The modeling and analysis of macromolecular structures has already made significant contributions to our understanding of how mutations affect the stability, folding, and binding of macromolecules [210]–[211]. Several recent structure-based studies have provided high-resolution pictures for how variants rewire biological networks through allosteric regulation, protein–protein interaction (PPI), and protein–nucleic acid interaction (PNI) [59], [140], [212]–[213]. Kowarsch et al. showed that co-evolving residues can influence each other through allosteric regulation and are significantly more likely to be disease-associated than expected by chance [140]. By mapping the mutations on the structures, Wang et al. found that in-frame mutations are enriched on the interaction interfaces of proteins associated with the corresponding diseases and that the disease specificity for different mutations can be explained by their location within an interface [59]. Similar findings were observed in David et al.'s work [213] by combining a database of the 3-D structures of human protein/protein complexes [214] and the humsavar database of nonsynonymous Single Nucleotide Polymorphisms (nsSNPs) [215].

As shown in Figure 1, proteome-wide drug–target interactions and genome-wide genetic variants may collectively affect the functional state of complex biological networks that mediate the system-level response to the drug, leading to both therapeutic and adverse effects. Few computational tools are able to model the collective effects of drug perturbation and genetic variants in the context of the whole human genome and biological network, which is essential for the development of personalized medicines. Pathway analysis such as gene-set enrichment analysis (GSEA) [216] can be applied to pharmacogenomics data to concentrate the genetic perturbation along the annotated biological pathways. Another way to use prior knowledge of gene interrelationships is to incorporate the information into the association study itself through Bayesian techniques [217] or by using boosting to prioritize disease networks [218]. By integrating the atomic details of molecular interactions, co-evolution information, protein–protein interaction networks, transcriptional profiles, and pathway enrichment analysis, Xie et al. developed a structural systems biology approach to identifying the functional role of DNA variants and causal mutations with an extremely small sample size [212]. Through this approach, the driver mutations that confer hypoxia tolerance in *Drosophila melanogaster* were identified. Furthermore, the functional roles of several nsSNPs, which are predominantly involved in allosteric regulation, protein–protein interaction, and protein–nucleic acid interaction, were determined [212]. The power of variation-mediated pathway analysis can be further enhanced by incorporating other regulatory and signaling network components, such as microRNA–target interactions, protein–nucleic acid interactions, and phosphorylation events, etc., and taking advantage of advanced graph mining algorithms [219]–[220].

The systematic perturbation of sequence variants can be introduced into the dynamics simulation of pathways and genome-scale modeling of biological networks through macromolecular structures. Recently, Cheng et al. developed a computational framework to integrate missense mutation, protein structural modeling, and ODE [221]. They introduced the Systemic Impact Factor (SIF) as a measurement of phenotype changes resulting from the mutation. SIF is a function of the free energy change caused by the mutation and systemic control coefficient. The free energy change in a protein directly leads to the change in its kinetic parameters. The control coefficient quantifies the sensitivity of the phenotype readout to the change in kinetic parameters. They tested their models on two cases: G2-M transition control in yeast and the human mitogen-activated protein kinase (MAPK) pathway. The SIF from the simulation is well correlated with the experimental results. The missense mutations in this study are mainly associated with protein stability. In the future, it will be interesting to quantify the systematic impact of broad types of mutations that modify allosteric regulation, molecular interaction, and gene expression. Chang et al. have integrated a reconstructed kidney model with structural bioinformatics and molecular modeling to predict the side effect profile of cholesterylester transfer protein (CETP) inhibitors and to identify genetic risk factors that cause adverse drug reactions [71]. An interesting finding from this study is the synthetic effect of drug–target interactions and nontarget-associated genetic modifications. Serious side effects are caused by the combination of the drug treatment and the genetic alteration but not by either alone. It is anticipated that the identification of nontarget genetic factors that affect drug action will have a significant impact on personalized medicine.

## Conclusion

The holistic, adaptive, and evolving nature of biological systems makes the quest for a simple and elegant mechanistic model to explain and predict biological phenomena less than fruitful. At the same time, the emergence of big data should significantly shift our approach to biomedical research. Given these data and increased computer power, useful predictive statistical models are possible. The question then becomes, how can we discover new knowledge from these statistical models under conditions that are constantly changing?

A complex disease state is not static under drug treatment, but evolves to new states in adapting to the drug-induced environment. When enough data are collected to describe one disease state and a successful model can be built, the disease may already be different from the one used to build the model. In such a temporal situation, a data-driven model is essentially retrospective but not prospective. Another major question is how much data are enough to build an accurate predictive model given the genetic, epigenetic, and clinical heterogeneity of complex diseases? Will the model still work if the individual has an unobserved new mutation? Moreover, genetic and/or epigenetic events and drug actions are rooted in the fundamental principle of physics and chemistry. Indifference to the detailed physical and chemical nature of biological processes in the modeling of big biological data could eventually hinder scientific advances in biomedicine. Discovery of new knowledge requires more than just a query of a big reference table built from data. Macromolecular structure plays an irreplaceable role in linking the physical and chemical origins of genetic events and drug action to the systematic response at the cellular, tissue, and organism levels. Thus, the incorporation of physiochemical-based macromolecular structure modeling with data-driven and mathematical-based pharmacodynamics, pharmacokinetics, pharmacogenomics, and systems pharmacology will not only enhance the power of modeling a predictive personalized drug response but will also shed new light on our understanding of living systems in a broad sense.

One of the barriers in applying macromolecular structure to pharmacogenomics and systems pharmacology is that the structural coverage of macromolecules and their complexes has been limited. Recent progress in both experimental and computational techniques has dramatically improved the structural coverage of the human genome. In addition to continuous efforts in structural genomics [222], breakthroughs in the crystallography of membrane proteins make representative 3-D structures of pharmaceutically important proteins such as G-protein coupled receptors (GPCR) [223] and transporters available [224]–[226]. Moreover, complexes of protein-ligand, protein-protein, and protein-nucleic acid structures, as found in the Research Collaboratory for Structural Bioinformatics (RCSB) Protein Data Bank (PDB), are increasing rapidly in both number and complexity [227]. Using the increased number of structural templates, and given the availability of high-performance computing, structure prediction has become routine for data from next generation sequencing. Even without structural templates, novel protein folds can be predicted with increasing accuracy [228]. Using co-evolutionary information from amino acid residues, high-quality structural models can now be built for proteins that are not amenable to conventional homology modeling or other methods of ab initio prediction [229]. Integrative modeling using different experimental techniques has contributed significantly in constructing macromolecular ensembles of unprecedented complexity [230]. Similarly, novel computational techniques have been developed to predict proteome-scale, 3-D interaction models of protein–protein interactions from medium to high resolution [50]–[52], [58]. Collectively, these advances provide new opportunities to use macromolecular structures in pharmacogenomics and systems pharmacology.

## References

[1] Jones SJ (1995) An update and lessons from whole-genome sequencing projects. Curr Opin Genet Dev 5: 349–353. 7549430 10.1016/0959-437x(95)80050-6

[2] Wang Z, Gerstein M, Snyder M (2009) RNA-Seq: a revolutionary tool for transcriptomics. Nat Rev Genet 10: 57–63. 19015660 10.1038/nrg2484 PMC2949280

[3] Park PJ (2009) ChIP-seq: advantages and challenges of a maturing technology. Nat Rev Genet 10: 669–680. 19736561 10.1038/nrg2641 PMC3191340

[4] Meissner A, Gnirke A, Bell GW, Ramsahoye B, Lander ES, et al (2005) Reduced representation bisulfite sequencing for comparative high-resolution DNA methylation analysis. Nucleic Acids Res 33: 5868–5877. 16224102 10.1093/nar/gki901 PMC1258174

[5] Boyle AP, Davis S, Shulha HP, Meltzer P, Margulies EH, et al (2008) High-resolution mapping and characterization of open chromatin across the genome. Cell 132: 311–322. 18243105 10.1016/j.cell.2007.12.014 PMC2669738

[6] Eggert US (2013) The why and how of phenotypic small-molecule screens. Nat Chem Biol 9: 206–209. 23508174 10.1038/nchembio.1206

[7] Schenone M, Dancik V, Wagner BK, Clemons PA (2013) Target identification and mechanism of action in chemical biology and drug discovery. Nat Chem Biol 9: 232–240. 23508189 10.1038/nchembio.1199 PMC5543995

[8] Guiguemde WA, Shelat AA, Bouck D, Duffy S, Crowther GJ, et al (2010) Chemical genetics of Plasmodium falciparum. Nature 465: 311–315. 20485428 10.1038/nature09099 PMC2874979

[9] Gamo FJ, Sanz LM, Vidal J, de Cozar C, Alvarez E, et al (2010) Thousands of chemical starting points for antimalarial lead identification. Nature 465: 305–310. 20485427 10.1038/nature09107

[10] Berger SI, Iyengar R (2009) Network analyses in systems pharmacology. Bioinformatics 25: 2466–2472. 19648136 10.1093/bioinformatics/btp465 PMC2752618

[11] Sorger PK, Allerheiligen SRB, Abernethy DR, Altman RB, Brouwer KLR, et al. (2011) Quantitative and Systems Pharmacology in the Post-genomic Era: New Approaches to Discovering Drugs and Understanding Therapeutic Mechanisms. Ward R, editor. NIH White Paper. Available: http://www.nigms.nih.gov/Training/Documents/SystemsPharmaWPSorger2011.pdf. Accessed 7 April 2014.

[12] Yang R, Niepel M, Mitchison TK, Sorger PK (2010) Dissecting variability in responses to cancer chemotherapy through systems pharmacology. Clin Pharmacol Ther 88: 34–38. 20520606 10.1038/clpt.2010.96 PMC2941986

[13] Hansen J, Zhao S, Iyengar R (2011) Systems pharmacology of complex diseases. Annals New York Acad Sci 1245: E1–5. 10.1111/j.1749-6632.2011.06382.x PMC3620720 22417173

[14] Wist AD, Berger SI, Iyengar R (2009) Systems pharmacology and genome medicine: a future perspective. Genome Med 1: 11. 19348698 10.1186/gm11 PMC2651594

[15] Zhao S, Iyengar R (2012) Systems pharmacology: network analysis to identify multiscale mechanisms of drug action. Annu Rev Pharmacol Toxicol 52: 505–521. 22235860 10.1146/annurev-pharmtox-010611-134520 PMC3619403

[16] Xie L, Kinnings SL, Bourne PE (2012) Novel Computational Approaches to Polypharmacology as a Means to Define Responses to Individual Drugs. Annu Rev Pharmacol Toxicol 52: 361–379. 22017683 10.1146/annurev-pharmtox-010611-134630

[17] Sun Y, Campisi J, Higano C, Beer TM, Porter P, et al (2012) Treatment-induced damage to the tumor microenvironment promotes prostate cancer therapy resistance through WNT16B. Nat Med 18: 1359–1368. 22863786 10.1038/nm.2890 PMC3677971

[18] Straussman R, Morikawa T, Shee K, Barzily-Rokni M, Qian ZR, et al (2012) Tumour micro-environment elicits innate resistance to RAF inhibitors through HGF secretion. Nature 487: 500–504. 22763439 10.1038/nature11183 PMC3711467

[19] Wilson ID (2009) Drugs, bugs, and personalized medicine: pharmacometabonomics enters the ring. Proc Natl Acad Sci U S A 106: 14187–14188. 19706501 10.1073/pnas.0907721106 PMC2732874

[20] Gutiu IA, Andries A, Mircioiu C, Radulescu F, Georgescu AM, et al (2010) Pharmacometabonomics, pharmacogenomics and personalized medicine. Rom J Intern Med 48: 187–191. 21428184

[21] Nicholson JK, Wilson ID, Lindon JC (2011) Pharmacometabonomics as an effector for personalized medicine. Pharmacogenomics 12: 103–111. 21174625 10.2217/pgs.10.157

[22] McMillin DW, Negri JM, Mitsiades CS (2013) The role of tumour-stromal interactions in modifying drug response: challenges and opportunities. Nat Rev Drug Discov 12: 217–228. 23449307 10.1038/nrd3870

[23] Karr JR, Sanghvi JC, Macklin DN, Gutschow MV, Jacobs JM, et al (2012) A whole-cell computational model predicts phenotype from genotype. Cell 150: 389–401. 22817898 10.1016/j.cell.2012.05.044 PMC3413483

[24] Krauss M, Schaller S, Borchers S, Findeisen R, Lippert J, et al (2012) Integrating cellular metabolism into a multiscale whole-body model. PLoS Comput Biol 8: e1002750. 23133351 10.1371/journal.pcbi.1002750 PMC3486908

[25] Gutenkunst RN, Waterfall JJ, Casey FP, Brown KS, Myers CR, et al (2007) Universally sloppy parameter sensitivities in systems biology models. PLoS Comput Biol 3: 1871–1878. 17922568 10.1371/journal.pcbi.0030189 PMC2000971

[26] Wang Y, Bolton E, Dracheva S, Karapetyan K, Shoemaker BA, et al (2010) An overview of the PubChem BioAssay resource. Nucleic Acids Res 38: D255–266. 19933261 10.1093/nar/gkp965 PMC2808922

[27] Li Q, Cheng T, Wang Y, Bryant SH (2010) PubChem as a public resource for drug discovery. Drug Discov Today 15: 1052–1057. 20970519 10.1016/j.drudis.2010.10.003 PMC3010383

[28] Gaulton A, Bellis LJ, Bento AP, Chambers J, Davies M, et al (2012) ChEMBL: a large-scale bioactivity database for drug discovery. Nucleic Acids Res 40: D1100–1107. 21948594 10.1093/nar/gkr777 PMC3245175

[29] Palsson B, Zengler K (2010) The challenges of integrating multi-omic data sets. Nat Chem Biol 6: 787–789. 20976870 10.1038/nchembio.462

[30] Joyce AR, Palsson BO (2006) The model organism as a system: integrating ‘omics’ data sets. Nat Rev Mol Cell Biol 7: 198–210. 16496022 10.1038/nrm1857

[31] Wiley HS (2011) Integrating multiple types of data for signaling research: challenges and opportunities. Sci Signal 4: pe9. 21325205 10.1126/scisignal.2001826

[32] Derry JM, Mangravite LM, Suver C, Furia MD, Henderson D, et al (2012) Developing predictive molecular maps of human disease through community-based modeling. Nat Genet 44: 127–130. 22281773 10.1038/ng.1089 PMC3643818

[33] Oku Y (2010) Future perspectives - proposal for Oxford Physiome Project. Adv Exp Med Biol 669: 57–60. 20217321 10.1007/978-1-4419-5692-7_12

[34] Noble D (2009) Systems biology, the Physiome Project and oriental medicine. J Physiol Sci 59: 249–251. 19340544 10.1007/s12576-009-0021-2 PMC10717787

[35] Hunter PJ, Crampin EJ, Nielsen PM (2008) Bioinformatics, multiscale modeling and the IUPS Physiome Project. Brief Bioinform 9: 333–343. 18477639 10.1093/bib/bbn024

[36] Nussinov R, Tsai CJ, Csermely P (2011) Allo-network drugs: harnessing allostery in cellular networks. Trends Pharmacol Sci 32: 686–693. 21925743 10.1016/j.tips.2011.08.004 PMC7380718

[37] White R, Peng G, Demir S (2009) Multiscale modeling of biomedical, biological, and behavioral systems (Part 1). IEEE Eng Med Biol Mag 28: 12–13. 19349247 10.1109/MEMB.2009.932388

[38] White RJ, Peng GC, Demir SS (2009) Multiscale modeling of biomedical, biological, and behavioral systems (part 2). IEEE Eng Med Biol Mag 28: 8–9. 19457728 10.1109/MEMB.2009.932490

[39] Dada JO, Mendes P (2011) Multi-scale modelling and simulation in systems biology. Integr Biol (Camb) 3: 86–96. 21212881 10.1039/c0ib00075b

[40] Flores SC, Bernauer J, Shin S, Zhou R, Huang X (2012) Multiscale modeling of macromolecular biosystems. Brief Bioinform 13: 395–405. 22228511 10.1093/bib/bbr077

[41] Silva JR, Pan H, Wu D, Nekouzadeh A, Decker KF, et al (2009) A multiscale model linking ion-channel molecular dynamics and electrostatics to the cardiac action potential. Proc Natl Acad Sci U S A 106: 11102–11106. 19549851 10.1073/pnas.0904505106 PMC2700153

[42] Obiol-Pardo C, Gomis-Tena J, Sanz F, Saiz J, Pastor M (2011) A multiscale simulation system for the prediction of drug-induced cardiotoxicity. J Chem Inf Model 51: 483–492. 21250697 10.1021/ci100423z

[43] Berger SI, Ma'ayan A, Iyengar R (2010) Systems pharmacology of arrhythmias. Sci Signal 3: ra30. 20407125 10.1126/scisignal.2000723 PMC3068558

[44] Xie L, Evangelidis T, Xie L, Bourne PE (2011) Drug Discovery Using Chemical Systems Biology: Weak inhibition of multiple kinases may contribute to the anti-cancer effect of Nelfinavir. PLoS Comp Biol 7: e1002037. 10.1371/journal.pcbi.1002037 PMC3084228 21552547

[45] Rico S, Antonijoan R, Barbanoj M (2009) Ebastine in the light of CONGA recommendations for the development of third-generation antihistamines. J Asthma Allergy 2: 73–92. 21437146 10.2147/jaa.s3108 PMC3048600

[46] Murphy RF (2011) An active role for machine learning in drug development. Nat Chem Biol 7: 327–330. 21587249 10.1038/nchembio.576 PMC4107394

[47] Chen B, Dong X, Jiao D, Wang H, Zhu Q, et al (2010) Chem2Bio2RDF: a semantic framework for linking and data mining chemogenomic and systems chemical biology data. BMC Bioinformatics 11: 255. 20478034 10.1186/1471-2105-11-255 PMC2881087

[48] Lemons NW, Hu B, Hlavacek WS (2011) Hierarchical graphs for rule-based modeling of biochemical systems. BMC Bioinformatics 12: 45. 21288338 10.1186/1471-2105-12-45 PMC3152790

[49] Ashby D (2006) Bayesian statistics in medicine: a 25 year review. Stat Med 25: 3589–3631. 16947924 10.1002/sim.2672

[50] Tuncbag N, Gursoy A, Nussinov R, Keskin O (2011) Predicting protein-protein interactions on a proteome scale by matching evolutionary and structural similarities at interfaces using PRISM. Nature Protocols 6: 1341–1354. 21886100 10.1038/nprot.2011.367 PMC7384353

[51] Zhang QC, Petrey D, Garzon JI, Deng L, Honig B (2013) PrePPI: a structure-informed database of protein-protein interactions. Nucleic Acids Res 41: D828–833. 23193263 10.1093/nar/gks1231 PMC3531098

[52] Zhang QC, Petrey D, Deng L, Qiang L, Shi Y, et al (2012) Structure-based prediction of protein-protein interactions on a genome-wide scale. Nature 490: 556–560. 23023127 10.1038/nature11503 PMC3482288

[53] Mosca R, Ceol A, Aloy P (2013) Interactome3D: adding structural details to protein networks. Nat Methods 10: 47–53. 23399932 10.1038/nmeth.2289

[54] Kim PM, Lu LJ, Xia Y, Gerstein MB (2006) Relating three-dimensional structures to protein networks provides evolutionary insights. Science 314: 1938–1941. 17185604 10.1126/science.1136174

[55] Kiel C, Beltrao P, Serrano L (2008) Analyzing protein interaction networks using structural information. Annu Rev Biochem 77: 415–441. 18304007 10.1146/annurev.biochem.77.062706.133317

[56] Kuzu G, Keskin O, Gursoy A, Nussinov R (2012) Constructing structural networks of signaling pathways on the proteome scale. Curr Opin Struct Biol 22: 367–377. 22575757 10.1016/j.sbi.2012.04.004

[57] Kar G, Keskin O, Nussinov R, Gursoy A (2012) Human proteome-scale structural modeling of E2-E3 interactions exploiting interface motifs. J Proteome Res 11: 1196–1207. 22149024 10.1021/pr2009143 PMC3285560

[58] Franzosa EA, Xia Y (2011) Structural principles within the human-virus protein-protein interaction network. Proc Natl Acad Sci U S A 108: 10538–10543. 21680884 10.1073/pnas.1101440108 PMC3127880

[59] Wang X, Wei X, Thijssen B, Das J, Lipkin SM, et al (2012) Three-dimensional reconstruction of protein networks provides insight into human genetic disease. Nat Biotechnol 30: 159–164. 22252508 10.1038/nbt.2106 PMC3708476

[60] Duran-Frigola M, Mosca R, Aloy P (2013) Structural systems pharmacology: the role of 3D structures in next-generation drug development. Chem Biol 20: 674–684. 23706634 10.1016/j.chembiol.2013.03.004

[61] Price ND, Reed JL, Palsson BO (2004) Genome-scale models of microbial cells: evaluating the consequences of constraints. Nat Rev Microbiol 2: 886–897. 15494745 10.1038/nrmicro1023

[62] Shen Y, Liu J, Estiu G, Isin B, Ahn YY, et al (2010) Blueprint for antimicrobial hit discovery targeting metabolic networks. Proc Natl Acad Sci U S A 107: 1082–1087. 20080587 10.1073/pnas.0909181107 PMC2824290

[63] Zhang Y, Thiele I, Weekes D, Li Z, Jaroszewski L, et al (2009) Three-dimensional structural view of the central metabolic network of Thermotoga maritima. Science 325: 1544–1549. 19762644 10.1126/science.1174671 PMC2833182

[64] Chang RL, Andrews K, Kim D, Li Z, Godzik A, et al (2013) Structural systems biology evaluation of metabolic thermotolerance in Escherichia coli. Science 340: 1220–1223. 23744946 10.1126/science.1234012 PMC3777776

[65] Chang RL, Xie L, Bourne PE, Palsson BO (2013) Antibacterial mechanisms identified through structural systems pharmacology. BMC Syst Biol 7: 102. 24112686 10.1186/1752-0509-7-102 PMC3853765

[66] Duarte NC, Becker SA, Jamshidi N, Thiele I, Mo ML, et al (2007) Global reconstruction of the human metabolic network based on genomic and bibliomic data. Proc Natl Acad Sci U S A 104: 1777–1782. 17267599 10.1073/pnas.0610772104 PMC1794290

[67] Bordbar A, Lewis NE, Schellenberger J, Palsson BO, Jamshidi N (2010) Insight into human alveolar macrophage and M. tuberculosis interactions via metabolic reconstructions. Mol Syst Biol 6: 422. 20959820 10.1038/msb.2010.68 PMC2990636

[68] Jerby L, Shlomi T, Ruppin E (2010) Computational reconstruction of tissue-specific metabolic models: application to human liver metabolism. Mol Syst Biol 6: 401. 20823844 10.1038/msb.2010.56 PMC2964116

[69] Shlomi T, Cabili MN, Herrgard MJ, Palsson BO, Ruppin E (2008) Network-based prediction of human tissue-specific metabolism. Nat Biotechnol 26: 1003–1010. 18711341 10.1038/nbt.1487

[70] Becker SA, Palsson BO (2008) Context-specific metabolic networks are consistent with experiments. PLoS Comput Biol 4: e1000082. 18483554 10.1371/journal.pcbi.1000082 PMC2366062

[71] Chang RL, Xie L, Xie L, Bourne PE, Palsson B (2010) Drug Off-Target Effects Predicted Using Structural Analysis in the Context of a Metabolic Network Model. PLoS Comput Biol 6: e1000938. 20957118 10.1371/journal.pcbi.1000938 PMC2950675

[72] Ng C, Hauptman R, Zhang YL, Bourne PE, Xie L (2014) Anti-infectious drug repurposing using an integrated chemical genomics and structural systems biology approach. Pac Symp Biocomput 19: 136–147. 24297541 PMC6322395

[73] Kinnings SL, Xie L, Fung K, Xie L, Bourne PE (2010) The Mycobacterium tuberculosis Drugome and Its Polypharmacological Implications. PLoS Comput Biol 6: e100976. 10.1371/journal.pcbi.1000976 PMC2973814 21079673

[74] Luo H, Chen J, Shi L, Mikailov M, Zhu H, et al (2011) DRAR-CPI: a server for identifying drug repositioning potential and adverse drug reactions via the chemical-protein interactome. Nucleic Acids Res 39: W492–498. 21558322 10.1093/nar/gkr299 PMC3125745

[75] Kufareva I, Ilatovskiy AV, Abagyan R (2012) Pocketome: an encyclopedia of small-molecule binding sites in 4D. Nucleic Acids Res 40: D535–540. 22080553 10.1093/nar/gkr825 PMC3245087

[76] Kalinina OV, Wichmann O, Apic G, Russell RB (2012) ProtChemSI: a network of protein-chemical structural interactions. Nucleic Acids Res 40: D549–553. 22110041 10.1093/nar/gkr1049 PMC3245083

[77] Nasr RJ, Swamidass SJ, Baldi PF (2009) Large scale study of multiple-molecule queries. J Cheminform 1: 7. 20298525 10.1186/1758-2946-1-7 PMC3225883

[78] Swamidass SJ, Azencott CA, Lin TW, Gramajo H, Tsai SC, et al (2009) Influence relevance voting: an accurate and interpretable virtual high throughput screening method. J Chem Inf Model 49: 756–766. 19391629 10.1021/ci8004379 PMC2750043

[79] Baldi P, Nasr R (2010) When is chemical similarity significant? The statistical distribution of chemical similarity scores and its extreme values. J Chem Inf Model 50: 1205–1222. 20540577 10.1021/ci100010v PMC2914517

[80] Keiser MJ, Roth BL, Armbruster BN, Ernsberger P, Irwin JJ, et al (2007) Relating protein pharmacology by ligand chemistry. Nat Biotechnol 25: 197–206. 17287757 10.1038/nbt1284

[81] Takarabe M, Kotera M, Nishimura Y, Goto S, Yamanishi Y (2012) Drug target prediction using adverse event report systems: a pharmacogenomic approach. Bioinformatics 28: i611–i618. 22962489 10.1093/bioinformatics/bts413 PMC3436840

[82] Yamanishi Y, Araki M, Gutteridge A, Honda W, Kanehisa M (2008) Prediction of drug-target interaction networks from the integration of chemical and genomic spaces. Bioinformatics 24: i232–240. 18586719 10.1093/bioinformatics/btn162 PMC2718640

[83] Nagamine N, Shirakawa T, Minato Y, Torii K, Kobayashi H, et al (2009) Integrating statistical predictions and experimental verifications for enhancing protein-chemical interaction predictions in virtual screening. PLoS Comput Biol 5: e1000397. 19503826 10.1371/journal.pcbi.1000397 PMC2685987

[84] Vina D, Uriarte E, Orallo F, Gonzalez-Diaz H (2009) Alignment-free prediction of a drug-target complex network based on parameters of drug connectivity and protein sequence of receptors. Mol Pharm 6: 825–835. 19281186 10.1021/mp800102c

[85] Gottlieb A, Stein GY, Ruppin E, Sharan R (2011) PREDICT: a method for inferring novel drug indications with application to personalized medicine. Mol Syst Biol 7: 496. 21654673 10.1038/msb.2011.26 PMC3159979

[86] Cheng F, Liu C, Jiang J, Lu W, Li W, et al (2012) Prediction of drug-target interactions and drug repositioning via network-based inference. PLoS Comput Biol 8: e1002503. 22589709 10.1371/journal.pcbi.1002503 PMC3349722

[87] Mei JP, Kwoh CK, Yang P, Li XL, Zheng J (2013) Drug-target interaction prediction by learning from local information and neighbors. Bioinformatics 29: 238–245. 23162055 10.1093/bioinformatics/bts670

[88] van Laarhoven T, Marchiori E (2013) Predicting Drug-Target Interactions for New Drug Compounds Using a Weighted Nearest Neighbor Profile. PLoS ONE 8: e66952. 23840562 10.1371/journal.pone.0066952 PMC3694117

[89] Alaimo S, Pulvirenti A, Giugno R, Ferro A (2013) Drug-target interaction prediction through domain-tuned network-based inference. Bioinformatics 29: 2004–2008. 23720490 10.1093/bioinformatics/btt307 PMC3722516

[90] Oprea TI, Nielsen SK, Ursu O, Yang JJ, Taboureau O, et al (2011) Associating Drugs, Targets and Clinical Outcomes into an Integrated Network Affords a New Platform for Computer-Aided Drug Repurposing. Mol Inform 30: 100–111. 22287994 10.1002/minf.201100023 PMC3266123

[91] Iacucci E, Tranchevent LC, Popovic D, Pavlopoulos GA, De Moor B, et al (2012) ReLiance: a machine learning and literature-based prioritization of receptor-ligand pairings. Bioinformatics 28: i569–i574. 22962483 10.1093/bioinformatics/bts391 PMC3436818

[92] Campillos M, Kuhn M, Gavin AC, Jensen LJ, Bork P (2008) Drug target identification using side-effect similarity. Science 321: 263–266. 18621671 10.1126/science.1158140

[93] Iskar M, Campillos M, Kuhn M, Jensen LJ, van Noort V, et al (2010) Drug-induced regulation of target expression. PLoS Comput Biol 6: e1000925. 20838579 10.1371/journal.pcbi.1000925 PMC2936514

[94] Chiang AP, Butte AJ (2009) Systematic evaluation of drug-disease relationships to identify leads for novel drug uses. Clin Pharmacol Ther 86: 507–510. 19571805 10.1038/clpt.2009.103 PMC2836384

[95] Spitzmuller A, Mestres J (2013) Prediction of the P. falciparum target space relevant to malaria drug discovery. PLoS Comput Biol 9: e1003257. 24146604 10.1371/journal.pcbi.1003257 PMC3798273

[96] Xie L, Xie L, Bourne PE (2011) Structure-based systems biology for analyzing off-target binding. Curr Opin Struct Biol 21: 189–199. 21292475 10.1016/j.sbi.2011.01.004 PMC3070778

[97] Martinez-Jimenez F, Papadatos G, Yang L, Wallace IM, Kumar V, et al (2013) Target prediction for an open access set of compounds active against Mycobacterium tuberculosis. PLoS Comput Biol 9: e1003253. 24098102 10.1371/journal.pcbi.1003253 PMC3789770

[98] Xie L, Li J, Xie L, Bourne PE (2009) Drug discovery using chemical systems biology: identification of the protein-ligand binding network to explain the side effects of CETP inhibitors. PLoS Comput Biol 5: e1000387. 19436720 10.1371/journal.pcbi.1000387 PMC2676506

[99] Garvie CW, Wolberger C (2001) Recognition of specific DNA sequences. Mol Cell 8: 937–946. 11741530 10.1016/s1097-2765(01)00392-6

[100] Rohs R, West SM, Sosinsky A, Liu P, Mann RS, et al (2009) The role of DNA shape in protein-DNA recognition. Nature 461: 1248–1253. 19865164 10.1038/nature08473 PMC2793086

[101] Rohs R, West SM, Liu P, Honig B (2009) Nuance in the double-helix and its role in protein-DNA recognition. Curr Opin Struct Biol 19: 171–177. 19362815 10.1016/j.sbi.2009.03.002 PMC2701566

[102] Eldar A, Elowitz MB (2010) Functional roles for noise in genetic circuits. Nature 467: 167–173. 20829787 10.1038/nature09326 PMC4100692

[103] Lemerle C, Di Ventura B, Serrano L (2005) Space as the final frontier in stochastic simulations of biological systems. FEBS Lett 579: 1789–1794. 15763553 10.1016/j.febslet.2005.02.009

[104] Aldridge BB, Burke JM, Lauffenburger DA, Sorger PK (2006) Physicochemical modelling of cell signalling pathways. Nat Cell Biol 8: 1195–1203. 17060902 10.1038/ncb1497

[105] Araujo RP, Petricoin EF, Liotta LA (2005) A mathematical model of combination therapy using the EGFR signaling network. Biosystems 80: 57–69. 15740835 10.1016/j.biosystems.2004.10.002

[106] Araujo RP, Liotta LA, Petricoin EF (2007) Proteins, drug targets and the mechanisms they control: the simple truth about complex networks. Nat Rev Drug Discov 6: 871–880. 17932492 10.1038/nrd2381

[107] Yang K, Bai H, Ouyang Q, Lai L, Tang C (2008) Finding multiple target optimal intervention in disease-related molecular network. Mol Syst Biol 4: 228. 18985027 10.1038/msb.2008.60 PMC2673713

[108] Iadevaia S, Lu Y, Morales FC, Mills GB, Ram PT (2010) Identification of optimal drug combinations targeting cellular networks: integrating phospho-proteomics and computational network analysis. Cancer Res 70: 6704–6714. 20643779 10.1158/0008-5472.CAN-10-0460 PMC2932856

[109] Qi X (2006) Stochastic models for prodrug targeting. 1. Diffusion of the efflux drug. Mol Pharm 3: 187–195. 16579648 10.1021/mp050089l

[110] Khalili S, Monaco JM, Armaou A (2010) Development of a stochastic model for the efficacy of NRTIs using known mechanisms of action. J Theor Biol 265: 704–717. 20510251 10.1016/j.jtbi.2010.05.006

[111] Stein M, Gabdoulline RR, Wade RC (2007) Bridging from molecular simulation to biochemical networks. Curr Opin Struct Biol 17: 166–172. 17395455 10.1016/j.sbi.2007.03.014

[112] Warshel A, Sharma PK, Kato M, Xiang Y, Liu H, et al (2006) Electrostatic basis for enzyme catalysis. Chem Rev 106: 3210–3235. 16895325 10.1021/cr0503106

[113] Wade RC, Gabdoulline RR, Ludemann SK, Lounnas V (1998) Electrostatic steering and ionic tethering in enzyme-ligand binding: insights from simulations. Proc Natl Acad Sci U S A 95: 5942–5949. 9600896 10.1073/pnas.95.11.5942 PMC34177

[114] Gabdoulline RR, Stein M, Wade RC (2007) qPIPSA: relating enzymatic kinetic parameters and interaction fields. BMC Bioinformatics 8: 373. 17919319 10.1186/1471-2105-8-373 PMC2174957

[115] Dell'Orco D (2009) Fast predictions of thermodynamics and kinetics of protein-protein recognition from structures: from molecular design to systems biology. Mol Biosyst 5: 323–334. 19396368 10.1039/b821580d

[116] Bai H, Yang K, Yu D, Zhang C, Chen F, et al (2011) Predicting kinetic constants of protein-protein interactions based on structural properties. Proteins 79: 720–734. 21287608 10.1002/prot.22904

[117] Moal IH, Bates PA (2012) Kinetic rate constant prediction supports the conformational selection mechanism of protein binding. PLoS Comput Biol 8: e1002351. 22253587 10.1371/journal.pcbi.1002351 PMC3257286

[118] Ren J, Xie L, Li WW, Bourne PE (2010) SMAP-WS: a parallel web service for structural proteome-wide ligand-binding site comparison. Nucleic Acids Res 38 Suppl: W441–444. 20484373 10.1093/nar/gkq400 PMC2896174

[119] Xie L, Bourne PE (2009) A unified statistical model to support local sequence order independent similarity searching for ligand-binding sites and its application to genome-based drug discovery. Bioinformatics 25: i305–312. 19478004 10.1093/bioinformatics/btp220 PMC2687974

[120] Xie L, Bourne PE (2008) Detecting evolutionary relationships across existing fold space, using sequence order-independent profile-profile alignments. Proc Natl Acad Sci U S A 105: 5441–5446. 18385384 10.1073/pnas.0704422105 PMC2291117

[121] Xie L, Bourne PE (2007) A robust and efficient algorithm for the shape description of protein structures and its application in predicting ligand binding sites. BMC Bioinformatics 8 Suppl 4: S9. 17570152 10.1186/1471-2105-8-S4-S9 PMC1892088

[122] Hermann JC, Marti-Arbona R, Fedorov AA, Fedorov E, Almo SC, et al (2007) Structure-based activity prediction for an enzyme of unknown function. Nature 448: 775–779. 17603473 10.1038/nature05981 PMC2254328

[123] Bulik S, Grimbs S, Huthmacher C, Selbig J, Holzhutter HG (2009) Kinetic hybrid models composed of mechanistic and simplified enzymatic rate laws–a promising method for speeding up the kinetic modelling of complex metabolic networks. FEBS J 276: 410–424. 19137631 10.1111/j.1742-4658.2008.06784.x

[124] Nam H, Lewis NE, Lerman JA, Lee DH, Chang RL, et al (2012) Network context and selection in the evolution to enzyme specificity. Science 337: 1101–1104. 22936779 10.1126/science.1216861 PMC3536066

[125] Wu Y, Vendome J, Shapiro L, Ben-Shaul A, Honig B (2011) Transforming binding affinities from three dimensions to two with application to cadherin clustering. Nature 475: 510–513. 21796210 10.1038/nature10183 PMC3167384

[126] Kenakin T, Christopoulos A (2013) Signalling bias in new drug discovery: detection, quantification and therapeutic impact. Nat Rev Drug Discov 12: 205–216. 23411724 10.1038/nrd3954

[127] Bruning JB, Parent AA, Gil G, Zhao M, Nowak J, et al (2010) Coupling of receptor conformation and ligand orientation determine graded activity. Nat Chem Biol 6: 837–843. 20924370 10.1038/nchembio.451 PMC2974172

[128] Kojetin DJ, Burris TP (2013) Small molecule modulation of nuclear receptor conformational dynamics: implications for function and drug discovery. Mol Pharmacol 83: 1–8. 22869589 10.1124/mol.112.079285 PMC3533479

[129] Amaro RE, Baron R, McCammon JA (2008) An improved relaxed complex scheme for receptor flexibility in computer-aided drug design. J Comput Aided Mol Des 22: 693–705. 18196463 10.1007/s10822-007-9159-2 PMC2516539

[130] Kobilka BK, Gether U (2002) Use of fluorescence spectroscopy to study conformational changes in the beta 2-adrenoceptor. Methods Enzymol 343: 170–182. 11665566 10.1016/s0076-6879(02)43134-5

[131] Hruby VJ, Tollin G (2007) Plasmon-waveguide resonance (PWR) spectroscopy for directly viewing rates of GPCR/G-protein interactions and quantifying affinities. Curr Opin Pharmacol 7: 507–514. 17869585 10.1016/j.coph.2007.08.001 PMC2151673

[132] Lohse MJ, Hein P, Hoffmann C, Nikolaev VO, Vilardaga JP, et al (2008) Kinetics of G-protein-coupled receptor signals in intact cells. Br J Pharmacol 153 Suppl 1: S125–132. 18193071 10.1038/sj.bjp.0707656 PMC2268076

[133] Baneres JL, Mesnier D, Martin A, Joubert L, Dumuis A, et al (2005) Molecular characterization of a purified 5-HT4 receptor: a structural basis for drug efficacy. J Biol Chem 280: 20253–20260. 15774473 10.1074/jbc.M412009200

[134] Okada T, Palczewski K (2001) Crystal structure of rhodopsin: implications for vision and beyond. Curr Opin Struct Biol 11: 420–426. 11495733 10.1016/s0959-440x(00)00227-x

[135] Pellissier LP, Sallander J, Campillo M, Gaven F, Queffeulou E, et al (2009) Conformational toggle switches implicated in basal constitutive and agonist-induced activated states of 5-hydroxytryptamine-4 receptors. Mol Pharmacol 75: 982–990. 19168624 10.1124/mol.108.053686

[136] Liu JJ, Horst R, Katritch V, Stevens RC, Wuthrich K (2012) Biased signaling pathways in beta2-adrenergic receptor characterized by 19F-NMR. Science 335: 1106–1110. 22267580 10.1126/science.1215802 PMC3292700

[137] Gelis L, Wolf S, Hatt H, Neuhaus EM, Gerwert K (2012) Prediction of a ligand-binding niche within a human olfactory receptor by combining site-directed mutagenesis with dynamic homology modeling. Angew Chem Int Ed Engl 51: 1274–1278. 22144177 10.1002/anie.201103980

[138] Taddese B, Simpson LM, Wall ID, Blaney FE, Kidley NJ, et al (2012) G-protein-coupled receptor dynamics: dimerization and activation models compared with experiment. Biochem Soc Trans 40: 394–399. 22435818 10.1042/BST20110755

[139] Li CY, Yu Q, Ye ZQ, Sun Y, He Q, et al (2007) A nonsynonymous SNP in human cytosolic sialidase in a small Asian population results in reduced enzyme activity: potential link with severe adverse reactions to oseltamivir. Cell Res 17: 357–362. 17426694 10.1038/cr.2007.27

[140] Kowarsch A, Fuchs A, Frishman D, Pagel P (2010) Correlated mutations: a hallmark of phenotypic amino acid substitutions. PLoS Comput Biol 6: e1000923. 20862353 10.1371/journal.pcbi.1000923 PMC2940720

[141] Lockless SW, Ranganathan R (1999) Evolutionarily conserved pathways of energetic connectivity in protein families. Science 286: 295–299. 10514373 10.1126/science.286.5438.295

[142] Dekker JP, Fodor A, Aldrich RW, Yellen G (2004) A perturbation-based method for calculating explicit likelihood of evolutionary co-variance in multiple sequence alignments. Bioinformatics 20: 1565–1572. 14962924 10.1093/bioinformatics/bth128

[143] Skerker JM, Perchuk BS, Siryaporn A, Lubin EA, Ashenberg O, et al (2008) Rewiring the specificity of two-component signal transduction systems. Cell 133: 1043–1054. 18555780 10.1016/j.cell.2008.04.040 PMC2453690

[144] Lee J, Natarajan M, Nashine VC, Socolich M, Vo T, et al (2008) Surface sites for engineering allosteric control in proteins. Science 322: 438–442. 18927392 10.1126/science.1159052 PMC3071530

[145] Lee SY, Banerjee A, MacKinnon R (2009) Two separate interfaces between the voltage sensor and pore are required for the function of voltage-dependent K(+) channels. PLoS Biol 7: e47. 19260762 10.1371/journal.pbio.1000047 PMC2650729

[146] Ferguson AD, Amezcua CA, Halabi NM, Chelliah Y, Rosen MK, et al (2007) Signal transduction pathway of TonB-dependent transporters. Proc Natl Acad Sci U S A 104: 513–518. 17197416 10.1073/pnas.0609887104 PMC1760641

[147] Zheng W, Brooks B (2005) Identification of dynamical correlations within the myosin motor domain by the normal mode analysis of an elastic network model. J Mol Biol 346: 745–759. 15713460 10.1016/j.jmb.2004.12.020

[148] Zheng W, Liao JC, Brooks BR, Doniach S (2007) Toward the mechanism of dynamical couplings and translocation in hepatitis C virus NS3 helicase using elastic network model. Proteins 67: 886–896. 17373706 10.1002/prot.21326

[149] Pan H, Lee JC, Hilser VJ (2000) Binding sites in Escherichia coli dihydrofolate reductase communicate by modulating the conformational ensemble. Proc Natl Acad Sci U S A 97: 12020–12025. 11035796 10.1073/pnas.220240297 PMC17287

[150] Chennubhotla C, Bahar I (2006) Markov propagation of allosteric effects in biomolecular systems: application to GroEL-GroES. Mol Syst Biol 2: 36. 16820777 10.1038/msb4100075 PMC1681507

[151] del Sol A, Fujihashi H, Amoros D, Nussinov R (2006) Residues crucial for maintaining short paths in network communication mediate signaling in proteins. Mol Syst Biol 2: 2006 0019. 16738564 10.1038/msb4100063 PMC1681495

[152] Saalau-Bethell SM, Woodhead AJ, Chessari G, Carr MG, Coyle J, et al (2012) Discovery of an allosteric mechanism for the regulation of HCV NS3 protein function. Nat Chem Biol 8: 920–925. 23023261 10.1038/nchembio.1081 PMC3480716

[153] Gleeson MP, Hersey A, Montanari D, Overington J (2011) Probing the links between in vitro potency, ADMET and physicochemical parameters. Nat Rev Drug Discov 10: 197–208. 21358739 10.1038/nrd3367 PMC6317702

[154] Copeland RA, Pompliano DL, Meek TD (2006) Drug-target residence time and its implications for lead optimization. Nat Rev Drug Discov 5: 730–739. 16888652 10.1038/nrd2082

[155] Lu H, Tonge PJ (2010) Drug-target residence time: critical information for lead optimization. Curr Opin Chem Biol 14: 467–474. 20663707 10.1016/j.cbpa.2010.06.176 PMC2918722

[156] Braz VA, Holladay LA, Barkley MD (2010) Efavirenz binding to HIV-1 reverse transcriptase monomers and dimers. Biochemistry 49: 601–610. 20039714 10.1021/bi901579y PMC2896556

[157] Lu H, England K, am Ende C, Truglio JJ, Luckner S, et al (2009) Slow-onset inhibition of the FabI enoyl reductase from francisella tularensis: residence time and in vivo activity. ACS Chem Biol 4: 221–231. 19206187 10.1021/cb800306y PMC2693246

[158] Copeland RA (2011) Conformational adaptation in drug-target interactions and residence time. Future Med Chem 3: 1491–1501. 21882942 10.4155/fmc.11.112

[159] Vauquelin G, Charlton SJ (2010) Long-lasting target binding and rebinding as mechanisms to prolong in vivo drug action. Br J Pharmacol 161: 488–508. 20880390 10.1111/j.1476-5381.2010.00936.x PMC2990149

[160] Carroll MJ, Mauldin RV, Gromova AV, Singleton SF, Collins EJ, et al (2012) Evidence for dynamics in proteins as a mechanism for ligand dissociation. Nat Chem Biol 8: 246–252. 22246400 10.1038/nchembio.769 PMC3288659

[161] ElSawy KM, Twarock R, Lane DP, Verma CS, Caves LSD (2012) Characterization of the Ligand Receptor Encounter Complex and Its Potential for in Silico Kinetics-Based Drug Development. J Chem Theory Comput 8: 314–321. 26592892 10.1021/ct200560w

[162] Laio A, Gervasio FL (2008) Metadynamics: a method to simulate rare events and reconstruct the free energy in biophysics, chemistry and material science. Rep Prog Phys 71.

[163] Pietrucci F, Marinelli F, Carloni P, Laio A (2009) Substrate Binding Mechanism of HIV-1 Protease from Explicit-Solvent Atomistic Simulations. J Am Chem Soc 131: 11811–11818. 19645490 10.1021/ja903045y

[164] Fitzgerald JB, Schoeberl B, Nielsen UB, Sorger PK (2006) Systems biology and combination therapy in the quest for clinical efficacy. Nat Chem Biol 2: 458–466. 16921358 10.1038/nchembio817

[165] Winter GE, Rix U, Carlson SM, Gleixner KV, Grebien F, et al (2012) Systems-pharmacology dissection of a drug synergy in imatinib-resistant CML. Nat Chem Biol 8: 905–912. 23023260 10.1038/nchembio.1085 PMC4038039

[166] Huang J, Niu C, Green CD, Yang L, Mei H, et al (2013) Systematic Prediction of Pharmacodynamic Drug-Drug Interactions through Protein-Protein-Interaction Network. PLoS Comput Biol 9: e1002998. 23555229 10.1371/journal.pcbi.1002998 PMC3605053

[167] Gottlieb A, Stein GY, Oron Y, Ruppin E, Sharan R (2012) INDI: a computational framework for inferring drug interactions and their associated recommendations. Mol Syst Biol 8: 592. 22806140 10.1038/msb.2012.26 PMC3421442

[168] Tatonetti NP, Ye PP, Daneshjou R, Altman RB (2012) Data-driven prediction of drug effects and interactions. Sci Transl Med 4: 125ra131. 10.1126/scitranslmed.3003377 PMC3382018 22422992

[169] Hansch C, Leo A, Mekapati SB, Kurup A (2004) QSAR and ADME. Bioorg Med Chem 12: 3391–3400. 15158808 10.1016/j.bmc.2003.11.037

[170] Hou T, Wang J, Zhang W, Wang W, Xu X (2006) Recent advances in computational prediction of drug absorption and permeability in drug discovery. Curr Med Chem 13: 2653–2667. 17017917 10.2174/092986706778201558

[171] Nettles JH, Jenkins JL, Williams C, Clark AM, Bender A, et al (2007) Flexible 3D pharmacophores as descriptors of dynamic biological space. J Mol Graph Model 26: 622–633. 17395510 10.1016/j.jmgm.2007.02.005

[172] Moroy G, Martiny VY, Vayer P, Villoutreix BO, Miteva MA (2012) Toward in silico structure-based ADMET prediction in drug discovery. Drug Discov Today 17: 44–55. 22056716 10.1016/j.drudis.2011.10.023

[173] Schlessinger A, Geier E, Fan H, Irwin JJ, Shoichet BK, et al (2011) Structure-based discovery of prescription drugs that interact with the norepinephrine transporter, NET. Proc Natl Acad Sci U S A 108: 15810–15815. 21885739 10.1073/pnas.1106030108 PMC3179104

[174] Wang Y, Xiao J, Suzek TO, Zhang J, Wang J, et al (2009) PubChem: a public information system for analyzing bioactivities of small molecules. Nucleic Acids Res 37: W623–633. 19498078 10.1093/nar/gkp456 PMC2703903

[175] Epstein SL, Yu X, Xie L (2013) Multi-agent, multi-case-based reasoning. Lecture Note in Comp Sci 7969: 74–88.

[176] Sun H, Scott DO (2010) Structure-based drug metabolism predictions for drug design. Chem Biol Drug Des 75: 3–17. 19878193 10.1111/j.1747-0285.2009.00899.x

[177] Nebert DW, Jorge-Nebert L, Vesell ES (2003) Pharmacogenomics and “individualized drug therapy”: high expectations and disappointing achievements. Am J Pharmacogenomics 3: 361–370. 14672516 10.2165/00129785-200303060-00002

[178] Zanger UM, Schwab M (2013) Cytochrome P450 enzymes in drug metabolism: Regulation of gene expression, enzyme activities, and impact of genetic variation. Pharmacol Ther 138: 103–141. 23333322 10.1016/j.pharmthera.2012.12.007

[179] Honkakoski P, Negishi M (2000) Regulation of cytochrome P450 (CYP) genes by nuclear receptors. Biochem J 347: 321–337. 10749660 10.1042/0264-6021:3470321 PMC1220963

[180] Scotto KW (2003) Transcriptional regulation of ABC drug transporters. Oncogene 22: 7496–7511. 14576854 10.1038/sj.onc.1206950

[181] Preissner S, Kroll K, Dunkel M, Senger C, Goldsobel G, et al (2010) SuperCYP: a comprehensive database on Cytochrome P450 enzymes including a tool for analysis of CYP-drug interactions. Nucleic Acids Res 38: D237–243. 19934256 10.1093/nar/gkp970 PMC2808967

[182] Zhang QY (2009) Genome-wide off-target binding of Rifampin and its implications for genetic disposition to drug toxicity. M.S. Thesis, The University of York.

[183] Nelson DR, Zeldin DC, Hoffman SM, Maltais LJ, Wain HM, et al (2004) Comparison of cytochrome P450 (CYP) genes from the mouse and human genomes, including nomenclature recommendations for genes, pseudogenes and alternative-splice variants. Pharmacogenetics 14: 1–18. 15128046 10.1097/00008571-200401000-00001

[184] Poliseno L (2012) Pseudogenes: newly discovered players in human cancer. Sci Signal 5: re5. 22990117 10.1126/scisignal.2002858

[185] Wang J, Pitarque M, Ingelman-Sundberg M (2006) 3′-UTR polymorphism in the human CYP2A6 gene affects mRNA stability and enzyme expression. Biochem Biophys Res Commun 340: 491–497. 16378601 10.1016/j.bbrc.2005.12.035

[186] Clayton TA, Baker D, Lindon JC, Everett JR, Nicholson JK (2009) Pharmacometabonomic identification of a significant host-microbiome metabolic interaction affecting human drug metabolism. Proc Natl Acad Sci U S A 106: 14728–14733. 19667173 10.1073/pnas.0904489106 PMC2731842

[187] Diaz Ochoa JG, Bucher J, Pery AR, Zaldivar Comenges JM, Niklas J, et al (2012) A multi-scale modeling framework for individualized, spatiotemporal prediction of drug effects and toxicological risk. Front Pharmacol 3: 204. 23346056 10.3389/fphar.2012.00204 PMC3551257

[188] Thorn CF, Klein TE, Altman RB (2005) PharmGKB: the pharmacogenetics and pharmacogenomics knowledge base. Methods Mol Biol 311: 179–191. 16100408 10.1385/1-59259-957-5:179

[189] Klein TE, Altman RB, Eriksson N, Gage BF, Kimmel SE, et al (2009) Estimation of the warfarin dose with clinical and pharmacogenetic data. N Engl J Med 360: 753–764. 19228618 10.1056/NEJMoa0809329 PMC2722908

[190] Garnett MJ, Edelman EJ, Heidorn SJ, Greenman CD, Dastur A, et al (2012) Systematic identification of genomic markers of drug sensitivity in cancer cells. Nature 483: 570–575. 22460902 10.1038/nature11005 PMC3349233

[191] Barretina J, Caponigro G, Stransky N, Venkatesan K, Margolin AA, et al (2012) The Cancer Cell Line Encyclopedia enables predictive modelling of anticancer drug sensitivity. Nature 483: 603–607. 22460905 10.1038/nature11003 PMC3320027

[192] Yeh CN, Chen TW, Lee HL, Liu YY, Chao TC, et al (2007) Kinase mutations and imatinib mesylate response for 64 Taiwanese with advanced GIST: preliminary experience from Chang Gung Memorial Hospital. Ann Surg Oncol 14: 1123–1128. 17195905 10.1245/s10434-006-9288-1

[193] Kobayashi S, Boggon TJ, Dayaram T, Janne PA, Kocher O, et al (2005) EGFR mutation and resistance of non-small-cell lung cancer to gefitinib. N Engl J Med 352: 786–792. 15728811 10.1056/NEJMoa044238

[194] Pao W, Miller VA, Politi KA, Riely GJ, Somwar R, et al (2005) Acquired resistance of lung adenocarcinomas to gefitinib or erlotinib is associated with a second mutation in the EGFR kinase domain. PLoS Med 2: e73. 15737014 10.1371/journal.pmed.0020073 PMC549606

[195] Mougey EB, Feng H, Castro M, Irvin CG, Lima JJ (2009) Absorption of montelukast is transporter mediated: a common variant of OATP2B1 is associated with reduced plasma concentrations and poor response. Pharmacogenet Genomics 19: 129–138. 19151602 10.1097/FPC.0b013e32831bd98c PMC2641037

[196] De Roock W, Jonker DJ, Di Nicolantonio F, Sartore-Bianchi A, Tu D, et al (2010) Association of KRAS p.G13D mutation with outcome in patients with chemotherapy-refractory metastatic colorectal cancer treated with cetuximab. JAMA 304: 1812–1820. 20978259 10.1001/jama.2010.1535

[197] Klein TE, Chang JT, Cho MK, Easton KL, Fergerson R, et al (2001) Integrating genotype and phenotype information: an overview of the PharmGKB project. Pharmacogenetics Research Network and Knowledge Base. Pharmacogenomics J 1: 167–170. 11908751 10.1038/sj.tpj.6500035

[198] Amado RG, Wolf M, Peeters M, Van Cutsem E, Siena S, et al (2008) Wild-type KRAS is required for panitumumab efficacy in patients with metastatic colorectal cancer. J Clin Oncol 26: 1626–1634. 18316791 10.1200/JCO.2007.14.7116

[199] Andre T, Blons H, Mabro M, Chibaudel B, Bachet JB, et al (2013) Panitumumab combined with irinotecan for patients with KRAS wild-type metastatic colorectal cancer refractory to standard chemotherapy: a GERCOR efficacy, tolerance, and translational molecular study. Ann Oncol 24: 412–419. 23041588 10.1093/annonc/mds465

[200] Abecasis GR, Auton A, Brooks LD, DePristo MA, Durbin RM, et al (2012) An integrated map of genetic variation from 1,092 human genomes. Nature 491: 56–65. 23128226 10.1038/nature11632 PMC3498066

[201] Dunham I, Kundaje A, Aldred SF, Collins PJ, Davis CA, et al (2012) An integrated encyclopedia of DNA elements in the human genome. Nature 489: 57–74. 22955616 10.1038/nature11247 PMC3439153

[202] Gerstein MB, Lu ZJ, Van Nostrand EL, Cheng C, Arshinoff BI, et al (2011) Integrative analysis of the Caenorhabditis elegans genome by the modENCODE project. Science 330: 1775–1787. 21177976 10.1126/science.1196914 PMC3142569

[203] Muers M (2011) Functional genomics: the modENCODE guide to the genome. Nat Rev Genet 12: 80. 21245826 10.1038/nrg2942

[204] Roy S, Ernst J, Kharchenko PV, Kheradpour P, Negre N, et al Identification of functional elements and regulatory circuits by Drosophila modENCODE. Science 330: 1787–1797. 21177974 10.1126/science.1198374 PMC3192495

[205] Davidson EH, Rast JP, Oliveri P, Ransick A, Calestani C, et al (2002) A genomic regulatory network for development. Science 295: 1669–1678. 11872831 10.1126/science.1069883

[206] Patwardhan RP, Hiatt JB, Witten DM, Kim MJ, Smith RP, et al (2012) Massively parallel functional dissection of mammalian enhancers in vivo. Nat Biotechnol 30: 265–270. 22371081 10.1038/nbt.2136 PMC3402344

[207] Sharon E, Kalma Y, Sharp A, Raveh-Sadka T, Levo M, et al (2012) Inferring gene regulatory logic from high-throughput measurements of thousands of systematically designed promoters. Nat Biotechnol 30: 521–530. 22609971 10.1038/nbt.2205 PMC3374032

[208] Melnikov A, Murugan A, Zhang X, Tesileanu T, Wang L, et al (2012) Systematic dissection and optimization of inducible enhancers in human cells using a massively parallel reporter assay. Nat Biotechnol 30: 271–277. 22371084 10.1038/nbt.2137 PMC3297981

[209] Kinney JB, Murugan A, Callan CG Jr, Cox EC (2010) Using deep sequencing to characterize the biophysical mechanism of a transcriptional regulatory sequence. Proc Natl Acad Sci U S A 107: 9158–9163. 20439748 10.1073/pnas.1004290107 PMC2889059

[210] Shi Z, Moult J (2011) Structural and functional impact of cancer-related missense somatic mutations. J Mol Biol 413: 495–512. 21763698 10.1016/j.jmb.2011.06.046 PMC4177034

[211] Wang Z, Moult J (2001) SNPs, protein structure, and disease. Human Mutation 17: 263–270. 11295823 10.1002/humu.22

[212] Xie L, Ng C, Ali T, Valencia R, Ferreira BL, et al (2013) Multiscale Modeling of the Causal Functional Roles of nsSNPs in a Genome-Wide Association Study: Application to Hypoxia. BMC Genomics 14: S9. 23819581 10.1186/1471-2164-14-S3-S9 PMC3665574

[213] David A, Razali R, Wass MN, Sternberg MJ (2012) Protein-protein interaction sites are hot spots for disease-associated nonsynonymous SNPs. Hum Mutat 33: 359–363. 22072597 10.1002/humu.21656

[214] Stein A, Mosca R, Aloy P (2011) Three-dimensional modeling of protein interactions and complexes is going 'omics. Curr Opin Struct Biol 21: 200–208. 21320770 10.1016/j.sbi.2011.01.005

[215] Stranger BE, Stahl EA, Raj T (2010) Progress and promise of genome-wide association studies for human complex trait genetics. Genetics 187: 367–383. 21115973 10.1534/genetics.110.120907 PMC3030483

[216] Subramanian A, Tamayo P, Mootha VK, Mukherjee S, Ebert BL, et al (2005) Gene set enrichment analysis: a knowledge-based approach for interpreting genome-wide expression profiles. Proc Natl Acad Sci U S A 102: 15545–15550. 16199517 10.1073/pnas.0506580102 PMC1239896

[217] Knight J, Barnes MR, Breen G, Weale ME (2011) Using functional annotation for the empirical determination of Bayes Factors for genome-wide association study analysis. PLoS ONE 6: e14808. 21556132 10.1371/journal.pone.0014808 PMC3083387

[218] Lee I, Blom UM, Wang PI, Shim JE, Marcotte EM (2011) Prioritizing candidate disease genes by network-based boosting of genome-wide association data. Genome Res 21: 1109–1121. 21536720 10.1101/gr.118992.110 PMC3129253

[219] Huang SS, Clarke DC, Gosline SJ, Labadorf A, Chouinard CR, et al (2013) Linking proteomic and transcriptional data through the interactome and epigenome reveals a map of oncogene-induced signaling. PLoS Comput Biol 9: e1002887. 23408876 10.1371/journal.pcbi.1002887 PMC3567149

[220] Huang SS, Fraenkel E (2009) Integrating proteomic, transcriptional, and interactome data reveals hidden components of signaling and regulatory networks. Sci Signal 2: ra40. 19638617 10.1126/scisignal.2000350 PMC2889494

[221] Cheng TM, Goehring L, Jeffery L, Lu YE, Hayles J, et al (2012) A structural systems biology approach for quantifying the systemic consequences of missense mutations in proteins. PLoS Comput Biol 8: e1002738. 23093928 10.1371/journal.pcbi.1002738 PMC3475653

[222] Dessailly BH, Nair R, Jaroszewski L, Fajardo JE, Kouranov A, et al (2009) PSI-2: structural genomics to cover protein domain family space. Structure 17: 869–881. 19523904 10.1016/j.str.2009.03.015 PMC2920419

[223] Stevens RC, Cherezov V, Katritch V, Abagyan R, Kuhn P, et al (2013) The GPCR Network: a large-scale collaboration to determine human GPCR structure and function. Nat Rev Drug Discov 12: 25–34. 23237917 10.1038/nrd3859 PMC3723354

[224] Jin MS, Oldham ML, Zhang Q, Chen J (2012) Crystal structure of the multidrug transporter P-glycoprotein from Caenorhabditis elegans. Nature 490: 566–569. 23000902 10.1038/nature11448 PMC3482266

[225] Korkhov VM, Mireku SA, Locher KP (2012) Structure of AMP-PNP-bound vitamin B12 transporter BtuCD-F. Nature 490: 367–372. 23000901 10.1038/nature11442

[226] Gopinath K, Venclovas C, Ioerger TR, Sacchettini JC, McKinney JD, et al (2013) A vitamin B(1)(2) transporter in Mycobacterium tuberculosis. Open Biol 3: 120175. 23407640 10.1098/rsob.120175 PMC3603451

[227] Berman HM, Coimbatore Narayanan B, Costanzo LD, Dutta S, Ghosh S, et al (2013) Trendspotting in the Protein Data Bank. FEBS Lett 587: 1036–1045. 23337870 10.1016/j.febslet.2012.12.029 PMC4068610

[228] Kryshtafovych A, Fidelis K, Moult J (2011) CASP9 results compared to those of previous CASP experiments. Proteins 79 Suppl 10: 196–207. 21997643 10.1002/prot.23182 PMC4180080

[229] Marks DS, Colwell LJ, Sheridan R, Hopf TA, Pagnani A, et al (2011) Protein 3D structure computed from evolutionary sequence variation. PLoS ONE 6: e28766. 22163331 10.1371/journal.pone.0028766 PMC3233603

[230] Ward AB, Sali A, Wilson IA (2013) Biochemistry. Integrative structural biology. Science 339: 913–915. 23430643 10.1126/science.1228565 PMC3633482

[231] Tseng YY, Chen ZJ, Li WH (2010) fPOP: footprinting functional pockets of proteins by comparative spatial patterns. Nucleic Acids Res 38: D288–295. 19880384 10.1093/nar/gkp900 PMC2808891

[232] Gao M, Skolnick J (2013) APoc: large-scale identification of similar protein pockets. Bioinformatics 29: 597–604. 23335017 10.1093/bioinformatics/btt024 PMC3582269

[233] Liu T, Altman RB (2011) Using multiple microenvironments to find similar ligand-binding sites: application to kinase inhibitor binding. PLoS Comput Biol 7: e1002326. 22219723 10.1371/journal.pcbi.1002326 PMC3248393

[234] Bryant DH, Moll M, Finn PW, Kavraki LE (2013) Combinatorial clustering of residue position subsets predicts inhibitor affinity across the human kinome. PLoS Comput Biol 9: e1003087. 23754939 10.1371/journal.pcbi.1003087 PMC3675009

[235] Milletti F, Vulpetti A (2010) Predicting polypharmacology by binding site similarity: from kinases to the protein universe. J Chem Inf Model 50: 1418–1431. 20666497 10.1021/ci1001263

[236] Sael L, Kihara D (2012) Detecting local ligand-binding site similarity in nonhomologous proteins by surface patch comparison. Proteins 80: 1177–1195. 22275074 10.1002/prot.24018 PMC3294165

[237] Ramensky V, Sobol A, Zaitseva N, Rubinov A, Zosimov V (2007) A novel approach to local similarity of protein binding sites substantially improves computational drug design results. Proteins 69: 349–357. 17623865 10.1002/prot.21487

[238] Xiong B, Wu J, Burk DL, Xue M, Jiang H, et al (2010) BSSF: a fingerprint based ultrafast binding site similarity search and function analysis server. BMC Bioinformatics 11: 47. 20100327 10.1186/1471-2105-11-47 PMC3098077

